# Total Synthesis of Xentrivalpeptide A and Its 6α-*epi* Derivative

**DOI:** 10.3390/molecules31183340

**Published:** 2026-09-20

**Authors:** Joonseong Hur, Yunseong Lee

**Affiliations:** College of Pharmacy and Inje Institute of Pharmaceutical Sciences and Research, Inje University, Gimhae-si 50834, Gyeongnam, Republic of Korea

**Keywords:** total synthesis, cyclodepsipeptide, xentrivalpeptide A, epimerization, macrocyclization

## Abstract

We report the first total synthesis of xentrivalpeptide A, a cyclic heptadepsipeptide natural product, through a convergent solution-phase strategy. The linear macrocyclization precursor was assembled from two peptide fragments, and a key DEPBT-mediated macrolactamization efficiently furnished the macrocyclic core despite the sterically demanding Val–Val cyclization junction. Late-stage installation of the side chain onto the common cyclic hexadepsipeptide core provides a potentially general route to other members of the xentrivalpeptide family. Additionally, switching the macrocyclization reagent from DEPBT to PyBOP enabled access to the diastereomeric congener 6α-*epi*-xentrivalpeptide A.

## 1. Introduction

Due to their ability to bind to undruggable protein surfaces, macrocycles have emerged as one of the most promising modalities in modern drug discovery [1,2]. Natural cyclic peptides represent a major class of bioactive macrocycles. The enormous structural diversity generated by nonribosomal peptides (NRPs) [3,4] and ribosomally synthesized and post-translationally modified peptides (RiPPs) [5] has led to clinically useful natural products, such as daptomycin and FK228, and continues to provide valuable templates for drug discovery.

**Figure 1:**
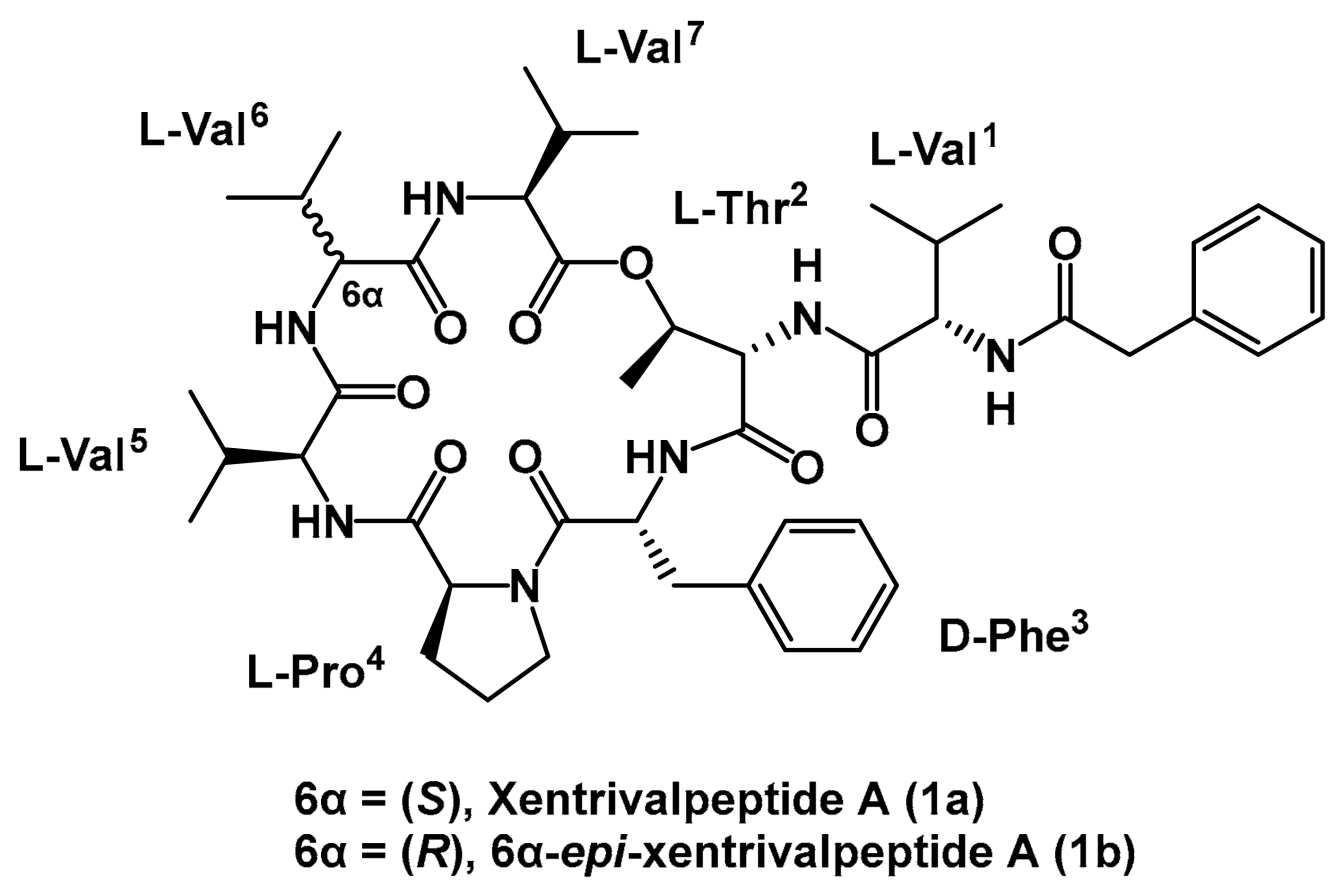
Structures of xentrivalpeptide A (**1a**) and 6α-*epi*-xentrivalpeptide A (**1b**).

In 2012, seventeen cyclodepsipeptides, collectively named xentrivalpeptides A–Q, were isolated from the entomopathogenic bacterium *Xenorhabdus* sp. by Bode et al. [6]. While the structure of a representative congener, xentrivalpeptide A (**1a**, Figure 1), was elucidated via NMR spectroscopy, that of other derivatives was indirectly determined based on isotope labeling and extensive mass analysis. Structurally, **1a** features a 19-membered macrocycle composed of 5 proteinogenic l-amino acids and 1 d-amino acid. The l-Thr^2^ amino acid in the macrocyclic backbone plays a pivotal role in the formation of depsipeptide linkage with l-Val^7^ and attachment of the *N*-acyl side chain, which is highly variable in nature. In terms of biological activity, only **1a** has been investigated because of the low natural production of the other derivatives (~1/100 compared to **1a**). **1a** exhibited no biological activity against standard test sets (antibacterial, antifungal, and cytotoxic tests) and only showed an actin cytoskeletal ruffling effect against insect hemocytes [6]. Because the biological function of xentrivalpeptides remains underexplored, versatile synthetic routes must be developed for this family.

Herein, we report the first total synthesis of xentrivalpeptide A (**1a**). Additionally, we describe the synthesis of the unnatural epimer 6α-*epi*-xentrivalpeptide A (**1b**), as a diastereomeric analog of the natural product.

## 2. Results and Discussion

**Scheme 1:**
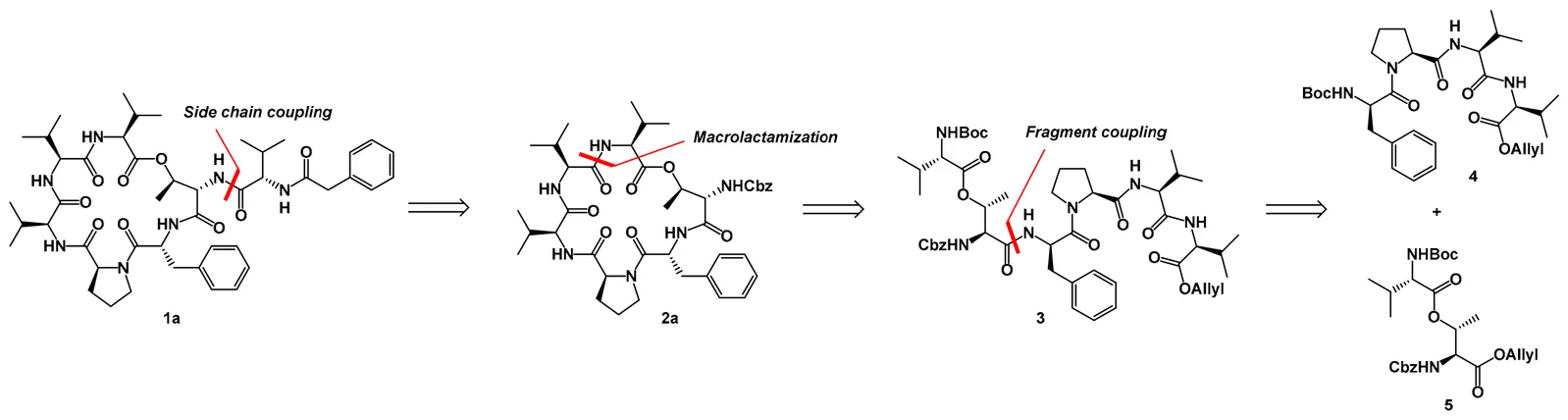
Retrosynthetic analysis of xentrivalpeptide A (**1a**).

Our retrosynthetic analysis of **1a** is outlined in Scheme 1. We conducted our synthesis in the conventional solution phase for the purpose of synthetic scalability. At first, we planned to install the side chain onto the common macrocyclic core **2a** at a late stage to enable facile synthesis of most members of the xentrivalpeptide series, which differ in the side chain amino acid and *N*-acyl endcap. Selection of the macrocyclization site requires careful consideration because there are no obvious cyclization sites such as glycine or alanine, which are sterically unhindered. For this reason, we considered the following criteria beyond steric hindrance: (1) macrolactonization between l-Thr^2^ and l-Val^7^ should be excluded due to the low nucleophilicity of the hydroxyl group, and (2) the labile ester bond, which may serve as a good leaving group [7] under basic conditions, should be formed as late as possible. Therefore, we envisioned that the amide bond between l-Val^6^ and l-Val^7^ could be a suitable macrocyclization site. By using this disconnection, the l-Pro^3^ residue, which is known to increase the probability of *β*-turn [8,9], could be located in the middle of the cyclization precursor. Hence, we also anticipated that this l-Pro^3^ residue may offer a beneficial effect during macrocyclization through turn-like preorganization, enabling the spatial proximity of both termini [10,11]. Lastly, the orthogonally protected linear depsipeptide **3** could be prepared via fragment coupling with tetrapeptide **4** and didepsipeptide **5**.

**Scheme 2:**
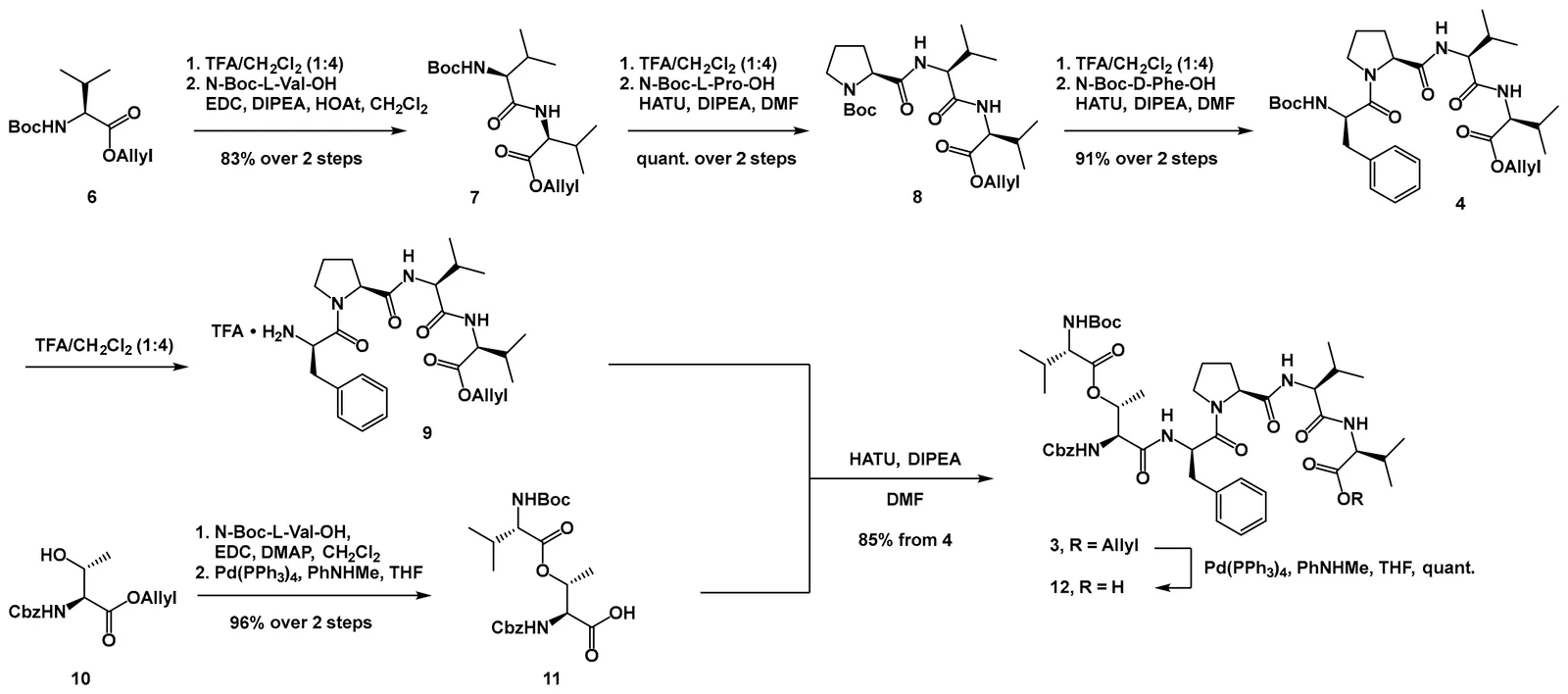
Synthesis of cyclization precursor **12**.

The synthesis of the macrocyclization precursor **12** commenced with the conventional peptide coupling strategy (Scheme 2). Starting from *N*-Boc-l-Val-OAllyl (**6**), an iterative Boc deprotection (using 20% TFA in CH_2_Cl_2_) and coupling cycle was performed to afford the desired tetrapeptide fragment **4** in 76% overall yield. Esterification of *N*-Cbz-L-Thr(OH)-OAllyl (**10**) with *N*-Boc-l-Val-OH, followed by Pd(0)-mediated allyl deprotection, afforded the didepsipeptide **11** at 96% over two steps. After Boc deprotection of **4**, the resulting tetrapeptide **9** was coupled with the didepsipeptide **11** to produce the hexadepsipeptide **3** in 85% yield, which was then subjected to macrocyclization. C-terminus deprotection was conducted to afford the carboxylic acid **12**.

With the *N*-protected linear precursor **12** in hand, we investigated suitable conditions for the key macrocyclization to furnish the cyclic hexadepsipeptide core **2a** (Table 1). For experimental convenience, an aliquot of the reaction mixture was dissolved in a solution containing a known amount of an internal peptide standard, and the reaction conversion was determined through HPLC analysis (see Supporting Information for details). Among the various combinations of coupling reagents and solvents tested, the DEPBT/DIPEA/DMF condition afforded the desired **2a** in the highest conversion ratio without any detectable epimerization (entry 8). The non-hydrogen-bonding solvent CH_2_Cl_2_ generally suppressed C-terminal epimerization compared with polar DMF (entries 1–4 vs. entries 5–9) [12]. Interestingly, PyBOP-mediated macrocyclization produced significant amounts of epimer **2b** in both solvents, as confirmed through LC–MS analysis (entries 3 and 7). The different extents of epimerization observed with different coupling reagents (e.g., DEPBT and PyBOP) are likely attributable to the structural differences in the corresponding activated ester intermediates [13,14], which could alter either their susceptibility to intramolecular nucleophilic attack or the local conformation around the C-terminal residue. These differences, in turn, may have affected the rate of oxazolone formation, a key pathway leading to epimerization [12,15]. When the cyclization precursor was slowly added to a solution of PyBOP and DIPEA in DMF over 12 h, diastereoselectivity was inverted, leading to the preferential formation of **2b** (entry 9). This change in diastereoselectivity may be attributed to the slow addition of the cyclization precursor, which increased the initial rate of C-terminal activation compared with the standard conditions (entry 7), thereby providing a greater chance for oxazolone formation before the precursor adopted a macrocyclization-favorable conformation. From a medicinal chemistry perspective, an epimeric congener of a cyclic peptide may adopt a distinct global conformation relative to its parent compound, potentially resulting in altered biological activity [16,17]. Therefore, we sought to exploit the epimerizing condition (e.g., entry 9) for the synthesis of 6α-*epi*-xentrivalpeptide A (**1b**).

**Scheme 3:**
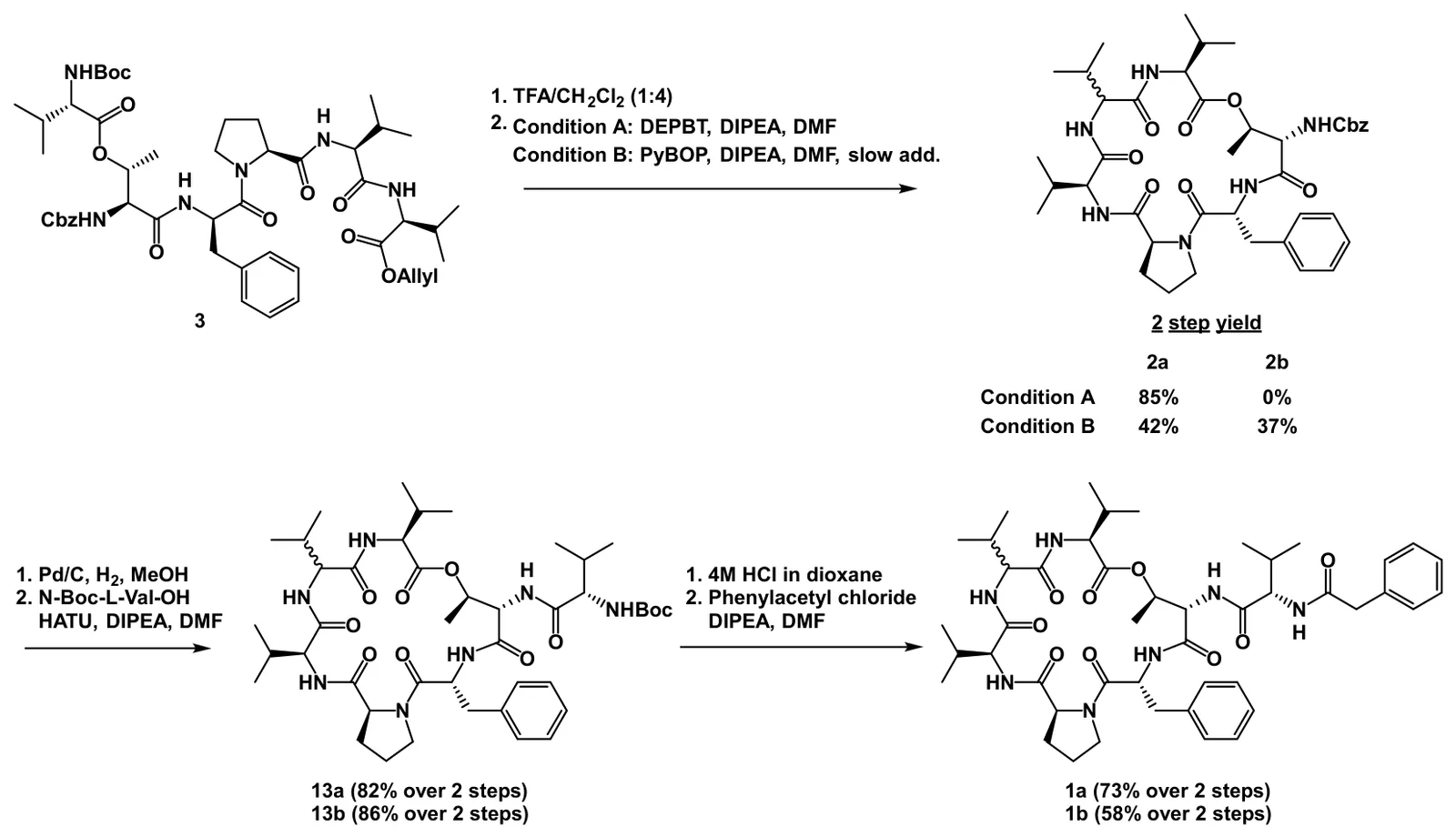
Synthesis of xentrivalpeptide A (**1a**) and 6α-*epi*-xentrivalpeptide A (**1b**).

**Figure 2:**
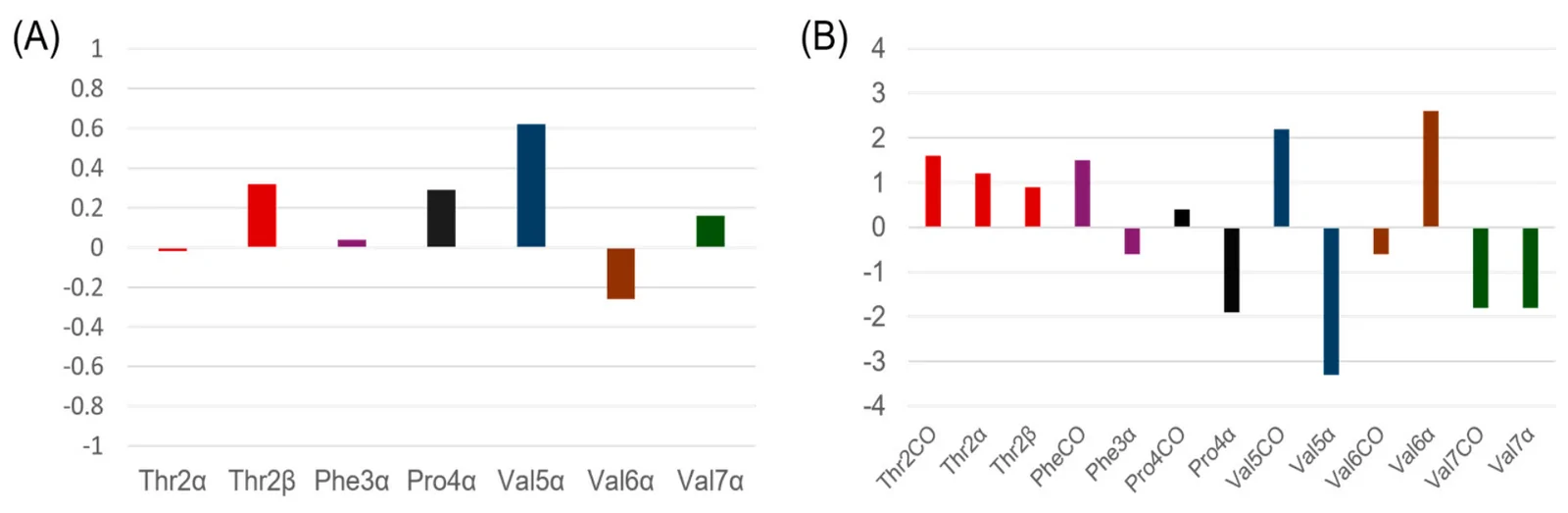
NMR chemical shift comparison between xentrivalpeptide A (**1a**) and 6α-*epi*-xentrivalpeptide A (**1b**). (**A**) Comparison of the proton chemical shifts. (**B**) Comparison of the carbon chemical shifts. The Y-axis represents Δ*δ* (**1b** − **1a**) in ppm.

Having established optimized macrocyclization conditions for **2a** and **2b**, we proceeded with the final side-chain installation onto the cyclic hexadepsipeptide core to complete the synthesis (Scheme 3). Following TFA-mediated Boc deprotection, treatment with 5 equivalents each of DEPBT and DIPEA at a substrate concentration of 1 mM in DMF (condition A) afforded the desired cyclic hexadepsipeptide **2a** in high yield (85% over two steps) without detectable epimerization. In contrast, PyBOP-mediated macrolactamization using a slow addition protocol for the linear substrate (condition B) afforded the 6α-*epi*-macrolactam **2b** in 37% yield, sufficient for further transformations, along with **2a** in 42% yield. The observed shift in the **2a**/**2b** selectivity may have arisen from the different reaction scales and concentration profiles employed in the screening and preparative reactions. Hydrogenolytic Cbz deprotection of **2a** and **2b**, followed by coupling with *N*-Boc-l-Val-OH, afforded the cyclic heptadepsipeptides **13a** and **13b** in 82% and 86% yields over two steps, respectively. Notably, no intramolecular *O*-to-*N* acyl shift was observed [18,19], although such a rearrangement could potentially occur through nucleophilic attack of the liberated amine on the depsipeptide ester bond following Cbz deprotection. This observation is likely due to the conformationally organized nature of the cyclic hexapeptide, which may increase the energetic barrier to the formation of a five-membered cyclic transition state leading to the acyl shift. This result indicates that our synthetic strategy should be amenable to facile side-chain diversification of xentrivalpeptide analogs. Finally, a second Boc deprotection followed by acylation with the phenylacetyl endcap successfully afforded the desired natural product **1a** and its epimer **1b** in 73% and 58% yields over two steps, respectively. All spectroscopic data (^1^H and ^13^C NMR and HRMS) of synthetic **1a** were fully consistent with the reported data (Table S1 in Supporting Information). The differences in the ^1^H and ^13^C NMR chemical shifts of the protons and carbons constituting the macrocyclic scaffold between **1a** and **1b** imply that epimerization at a single stereogenic center leads to an overall conformational change in the macrocycle (Figure 2; see Table S2 in the Supporting Information for details).

## 3. Materials and Methods

### 3.1. General Information

Methylene chloride (DCM) and tetrahydrofuran (THF) were dehydrated using molecular sieve (4 Å, beads, 4–8 mesh, Sigma Aldrich, St. Luis, MO, USA) pre-activated under 100 °C. All other solvents and reagents were purchased from commercial sources and used as received. Amino acid and coupling reagents were purchased from Sigma Aldrich, TCI, ChemeScene (Monmouth Junction, NJ, USA), Combi-blocks (San Diego, CA, USA) and BLDpharm (Shanghai, China). *N*,*N*-diisopropylethylamine (DIPEA) was purchased from SAMCHUN (Pyeongtaek, Republic of Korea). Dimethylformamide (DMF) were purchased from Sigma Aldrich in a Sure/Seal^TM^ bottle and used as received. Reactions were performed under an argon atmosphere with dry solvents under anhydrous conditions unless otherwise noted. Flash column chromatography was carried out using ZEOprep 60 Å 230–400 mesh silica gel. Thin-layer chromatography (TLC) was performed on Merck glass-backed TLC Silica gel 60 F_254_ and visualized using a UV lamp (254 nm), KMnO_4_, ninhydrin, anis aldehyde or Hanessian’s stain. ^1^H and ^13^C spectra were recorded on Varian 500 MHz spectrometers. ^1^H NMR spectra were referenced to CDCl_3_ (*δ* 7.26 ppm) DMSO-*d*_6_ (*δ* 2.50 ppm) and CD_3_OD (*δ* 3.30 ppm). ^13^C NMR spectra were referenced to CDCl_3_ (*δ* 77.10 ppm), DMSO-*d*_6_ (*δ* 39.52 ppm) and CD_3_OD (*δ* 49.15 ppm). Spectral data is reported as follows: chemical shift (in ppm), multiplicity (s = singlet, d = doublet, t = triplet, q = quartet, dd = doublet of doublets, dt = doublet of triplets, ddt = doublet of doublet of triplets, dtd = doublet of triplet of doublets, m = multiplet, br = broad), coupling constant (*J*) in Hertz (Hz), and integration. High resolution mass spectra were obtained on a JEOL JMS-700 (double focusing) mass spectrometer (JEOL, Tokyo, Japan). Optical rotations were measured with JASCO P-2000 digital polarimeter (JASCO, Tokyo, Japan) at 25 °C using cylindrical cell of 100 mm pathlength. Melting points were determined using a melting point apparatus MP50 by Mettler Toledo and are uncorrected (Mettler Toledo, Greifensee, Switzerland). Analytical HPLC measurments were performed for determination of compound purity and optimization of macrocyclization using a YL9100 system (YOUNGIN Chromass, Anyang, Republic of Korea). Low-resolution mass spectra (ESI) were collected on an Agilent Technologies 1200 series HPLC paired to a 6130 Mass Spectrometer (Agilent, SantaClara, CA, USA). Compounds were resolved on YMC-Triart C18 4.6 mm I.D. × 250 mm S-5 12 nm column at 30 °C with a flow of 1 mL/min. The gradient consisted of eluents A (0.1% formic acid in HPLC-grade acetonitrile) and eluents B (0.1% formic acid in double distilled water). A linear gradient starting from 30% of A to 95% over 20 min at a flow rate of 1.0 mL/min. Stays constant at 95% for 5 min, then returns to 30% over 0.5 min and stays constant at 30% for 5 min.

### 3.2. Experimental Procedures and Characterization of Compounds

allyl (*tert*-butoxycarbonyl)-L-valyl-L-valinate (**7**) To a solution of *N*-Boc-l-Val-OAllyl **6** (1.72 g, 6.68 mmol) in CH_2_Cl_2_ (28 mL) was added TFA (7 mL) dropwise at 0 °C. After stirring for 2 h at room temperature, the reaction mixture was concentrated in vacuo and dried thrice azeotropically with *n*-heptane. The amine-TFA salt was used in the next step without further purification. To a solution of the above amine (6.68 mmol), *N*-Boc-l-Val-OH (2.18 g, 10.02 mmol), HOAt (909 mg, 6.68 mmol) and DIPEA (3.5 mL, 20.04 mmol) in CH_2_Cl_2_ (33 mL) was added EDC (3.84 g, 20.04 mmol) at 0 °C. After stirring overnight at room temperature, the reaction mixture was quenched with 1 M HCl and extracted with CH_2_Cl_2_. The combined organic layer was washed with saturated NaHCO_3_, dried over MgSO_4_ and concentrated in vacuo. The residue was purified by flash column chromatography on silica gel (EtOAc/Hexane = 1:5) to give 1.97 g (83% over 2 steps) of dipeptide **7** as a white solid.

^1^H NMR (500 MHz, CDCl_3_) *δ* 6.35 (d, *J* = 8.8 Hz, 1H), 5.95–5.84 (m, 1H), 5.33 (dd, J = 17.2, 1.5 Hz, 1H), 5.26 (dd, J = 10.4, 1.3 Hz, 1H), 5.05 (d, J = 8.8 Hz, 1H), 4.68–4.59 (m, 2H), 4.57 (dd, J = 8.8, 4.8 Hz, 1H), 3.91 (dd, J = 8.8, 6.4 Hz, 1H), 2.26–2.16 (m, 1H), 2.15–2.07 (m, 1H), 1.44 (s, 9H), 0.99–0.89 (m, 12H).
^13^C NMR (126 MHz, CDCl_3_) *δ* 171.7, 171.4, 155.9, 131.6, 119.1, 80.0, 65.9, 60.2, 57.1, 31.3, 30.7, 28.4, 19.4, 19.0, 18.0, 17.7.
HRMS (FAB+) calcd for C_18_H_33_N_2_O_5_ (M+H^+^) 357.2389, found 357.2385.
[α]^25^_D_ = −24.1 (*c* 1.0, MeOH)

allyl (*tert*-butoxycarbonyl)-L-prolyl-L-valyl-L-valinate (**8**) To a solution of dipeptide **7** (1.45 g, 4.07 mmol) in CH_2_Cl_2_ (16 mL) was added TFA (4 mL) dropwise at 0 °C. After stirring for 1.5 h at room temperature, the reaction mixture was concentrated in vacuo and dried thrice azeotropically with *n*-heptane. The amine-TFA salt was used in the next step without further purification. To a solution of the above amine (4.07 mmol), *N*-Boc-l-Pro-OH (1.32 g, 6.11 mmol) and DIPEA (1.4 mL, 8.14 mmol) in CH_2_Cl_2_ (41 mL) was added HATU (3.10 g, 8.14 mmol) at 0 °C. After stirring overnight at room temperature, the reaction mixture was quenched with 1 M HCl and extracted with EtOAc. The combined organic layer was washed with saturated NaHCO_3_, dried over MgSO_4_ and concentrated in vacuo. The residue was purified by flash column chromatography on silica gel (EtOAc/Hexane = 2:3) to give 1.90 g (quant. over 2 steps) of tripeptide **8** as a colorless oil.

^1^H NMR (500 MHz, DMSO-*d*_6_, a mixture of rotamer) *δ* 8.20 (d, *J* = 7.7 Hz, 0.7H), 8.16 (d, *J* = 7.8 Hz, 0.3H), 7.67 (d, *J* = 8.8 Hz, 2H), 5.94–5.82 (m, 1H), 5.32 (dq, *J* = 17.2, 1.6 Hz, 1H), 5.21 (dd, *J* = 10.5, 1.6 Hz, 1H), 4.56 (dt, *J* = 5.5, 1.5 Hz, 2H), 4.31–4.23 (m, 1H), 4.22–4.13 (m, 2H), 3.40–3.30 (m, 1H, overlapped), 3.29–3.21 (m, 1H), 2.14–2.00 (m, 1H), 2.00–1.89 (m, 1H), 1.86–1.68 (m, 4H), 1.39 (s, 3H), 1.31 (s, 6H), 0.96–0.77 (m, 12H).
^13^C NMR (126 MHz, CDCl_3_, a mixture of rotamer) Major rotamer *δ* 172.2, 171.5, 170.9, 153.4, 132.3, 118.0, 78.4, 64.7, 59.4, 57.5, 57.1, 46.5, 31.1, 30.7, 29.7, 28.0, 22.9, 18.9, 18.2.
HRMS (FAB+) calcd for C_23_H_40_N_3_O_6_ (M+H^+^) 454.2917, found 454.2916.
[α]^25^_D_ = −40.2 (*c* 1.0, MeOH)

allyl (*tert*-butoxycarbonyl)-D-phenylalanyl-L-prolyl-L-valyl-L-valinate (**4**) To a solution of tripeptide **8** (167 mg, 0.37 mmol) in CH_2_Cl_2_ (6.0 mL) was added TFA (1.5 mL) dropwise at 0 °C. After stirring for 6 h at room temperature, the reaction mixture was concentrated in vacuo and dried thrice azeotropically with *n*-heptane. The amine-TFA salt was used in the next step without further purification. To a solution of the above amine (0.37 mmol), *N*-Boc-d-Phe-OH (149 mg, 0.56 mmol) and DIPEA (0.13 mL, 0.74 mmol) in DMF (7.4 mL) was added HATU (281 mg, 0.74 mmol) at 0 °C. After stirring overnight at room temperature, the reaction mixture was quenched with 1 M HCl and extracted with EtOAc. The combined organic layer was washed with saturated NaHCO_3_, dried over MgSO_4_ and concentrated in vacuo. The residue was purified by flash column chromatography on silica gel (EtOAc/Hexane = 1:1 to 3:2) to give 202 mg (91% over 2 steps) of tetrapeptide **4** as a colorless oil.

^1^H NMR (500 MHz, CDCl_3_) *δ* 7.46 (d, *J* = 8.4 Hz, 1H), 7.32–7.18 (m, 5H), 6.70 (d, *J* = 8.5 Hz, 1H), 5.96–5.84 (m, 1H), 5.33 (dd, J = 17.2, 1.5 Hz, 1H), 5.25 (d, J = 10.6 Hz, 1H), 4.68–4.55 (m, 3H), 4.52 (dd, *J* = 8.6, 4.9 Hz, 1H), 4.45 (d, *J* = 8.2 Hz, 1H), 4.14 (dd, *J* = 8.4, 6.3 Hz, 1H), 3.54 (t, *J* = 9.0 Hz, 1H), 3.08–2.90 (m, 2H), 2.52 (q, *J* = 8.8 Hz, 1H), 2.32–2.25 (m, 1H), 2.25–2.14 (m, 2H), 1.82–1.78 (m, 2H), 1.61–1.50 (m, 1H), 1.49–1.43 (m, 1H), 1.40 (s, 9H), 0.95 (d, *J* = 6.9 Hz, 3H), 0.93 (d, *J* = 6.8 Hz, 3H), 0.89 (d, *J* = 6.8 Hz, 6H).
^13^C-NMR (126 MHz, CDCl_3_) *δ* 172.2, 171.4, 171.2, 171.1, 155.2, 136.0, 131.8, 129.4, 128.7, 127.3, 118.9, 80.2, 65.8, 60.2, 59.4, 57.3, 53.8, 47.1, 39.6, 31.2, 29.8, 28.4, 27.5, 24.4, 19.6, 19.1, 17.9, 17.9.
HRMS (FAB+) calcd for C_32_H_49_N_4_O_7_ (M+H^+^) 601.3601, found 601.3597.
[α]^25^_D_ = −47.2 (*c* 0.5, MeOH)

allyl *N-*((benzyloxy)carbonyl)-*O*-((*tert*-butoxycarbonyl)-L-valyl)-L-threoninate (**5**) To a solution of *N*-Cbz-l-Thr-OAllyl **10** (522 mg, 1.79 mmol) and *N*-Boc-l-Val-OH (584 mg, 2.69 mmol) in CH_2_Cl_2_ (9 mL) was added EDC (686 mg, 3.58 mmol) and DMAP (437 mg, 3.58 mmol) at 0 °C. After stirring overnight at room temperature, the reaction mixture was quenched with 1 M HCl and extracted with CH_2_Cl_2_. The combined organic layer was washed with saturated NaHCO_3_, dried over MgSO_4_ and concentrated in vacuo. The residue was purified by flash column chromatography on silica gel (EtOAc/Hexane = 1:5) to give 846 mg (96%) of didepsipeptide **5** as a colorless oil.

^1^H NMR (500 MHz, DMSO-*d*_6_) *δ* 7.78 (d, *J* = 9.6 Hz, 1H), 7.42–7.29 (m, 5H), 7.14 (d, *J* = 9.3 Hz, 1H), 5.92–5.80 (m, 1H), 5.35–5.26 (m, 2H), 5.20 (dq, *J* = 10.5, 1.5 Hz, 1H), 5.16–5.04 (m, 2H), 4.55 (dt, *J* = 5.4, 1.5 Hz, 2H), 4.49 (dd, *J* = 9.6, 2.7 Hz, 1H), 3.78 (dd, *J* = 9.3, 7.2 Hz, 1H), 1.87 (h, *J* = 6.8 Hz, 1H), 1.36 (s, 9H), 1.16 (d, *J* = 6.3 Hz, 3H), 0.80 (d, *J* = 6.8 Hz, 3H), 0.77 (d, *J* = 6.8 Hz, 3H).
^13^C NMR (126 MHz, DMSO-*d*_6_) *δ* 170.5, 169.7, 157.2, 156.1, 137.2, 132.5, 128.9, 128.4, 128.4, 118.5, 78.8, 70.4, 66.4, 65.8, 60.2, 57.9, 30.6, 28.6, 19.3, 18.6, 16.8.
HRMS (FAB+) calcd for C_25_H_37_N_2_O_8_ (M+H^+^) 493.2550, found 493.2548.
[α]^25^_D_ = −1.0 (*c* 1.0, MeOH)

*N*-((benzyloxy)carbonyl)-*O*-((tert-butoxycarbonyl)-L-valyl)-L-threonine (**11**) To a solution of didepsipeptide **5** (305 mg, 0.62 mmol) in dry THF (6.2 mL), Pd(PPh_3_)_4_ (72 mg, 0.06 mmol) and *N*-methylaniline (0.13 mL, 1.24 mmol) was added at room temperature. After stirring for 1.5 h, the reaction mixture was quenched with 1 M HCl and extracted with EtOAc. The combined organic layer was dried over MgSO_4_ and concentrated in vacuo. The residue was purified by column chromatography on a short pad of silica gel (CH_2_Cl_2_/MeOH = 10:1) to afford 285 mg (quant.) of the acid **11** as a colorless oil.

^1^H NMR (500 MHz, DMSO-*d*_6_) *δ* 7.45–7.26 (m, 5H), 7.11 (d, *J* = 9.2 Hz, 1H), 5.33–5.23 (m, 1H), 5.08 (s, 2H), 4.23 (d, *J* = 9.5 Hz, 1H), 3.79 (t, *J* = 8.1 Hz, 1H), 1.96–1.86 (m, 1H), 1.36 (s, 9H), 1.13 (d, *J* = 6.2 Hz, 3H), 0.80 (dd, *J* = 6.8, 4.0 Hz, 6H).
^13^C NMR (126 MHz, DMSO-*d*_6_) *δ* 171.1, 170.2, 156.6, 155.7, 136.9, 128.4, 127.9, 78.3, 65.7, 59.8, 31.3, 30.2, 28.2, 22.1, 19.0, 18.1, 16.9, 14.0.
HRMS (FAB-) calcd for C_22_H_31_N_2_O_8_ (M−H^+^) 451.2081, found 450.2080.
[α]^25^_D_ = +5.1 (*c* 1.0, MeOH)

allyl *N*-((benzyloxy)carbonyl)-*O*-((tert-butoxycarbonyl)-L-valyl)-L-threonyl-D-phenylalanyl-L-prolyl-L-valyl-L-valinate (**3**) To a solution of tetrapeptide **4** (240 mg, 0.40 mmol) in CH_2_Cl_2_ (4 mL) was added TFA (1 mL) dropwise at 0 °C. After stirring for 2 h at room temperature, the reaction mixture was concentrated in vacuo, and dried thrice azeotropically with *n*-heptane. The amine-TFA salt was used in the next step without further purification. To the above amine (0.40 mmol), the didepsipeptide **11** (285 mg, 0.63 mmol) solution dissolved in anhydrous DMF (4 mL) was added. Subsequently, DIPEA (0.21 mL, 1.20 mmol) and HATU (456 mg, 1.20 mmol) were added to the above solution at 0 °C. After stirring overnight at room temperature, the reaction mixture was quenched with 1 M HCl and extracted with EtOAc. The combined organic layer was washed with saturated NaHCO_3_, dried over MgSO_4_ and concentrated in vacuo. The residue was purified by flash column chromatography on silica gel (EtOAc/Hexane = 3:2) to give 319 mg (85% over 2 steps) of hexadepsipeptide **3** as a clear caramel.

^1^H NMR (500 MHz, DMSO-*d*_6_) a mixture of rotamers *δ* 8.48 (d, *J* = 7.8 Hz, 0.5H), 8.32 (d, *J* = 8.7 Hz, 0.5H), 8.19 (d, *J* = 7.7 Hz, 0.5H), 8.14 (d, *J* = 8.5 Hz, 0.5H), 8.04 (d, *J* = 7.9 Hz, 0.5H), 7.78 (d, *J* = 8.8 Hz, 0.5H), 7.47 (d, *J* = 9.6 Hz, 0.5H), 7.40–7.27 (m, 5H), 7.27–7.18 (m, 2H), 7.18–7.10 (m, 33H), 7.10–7.05 (m, 1.5H), 5.94–5.82 (m, 1H), 5.32 (dtd, *J* = 17.2, 3.3, 1.7 Hz, 1H), 5.20 (ddq, *J* = 10.5, 3.0, 1.4 Hz, 1H), 5.15–4.99 (m, 2.5H), 4.92–4.84 (m, 1H), 4.69 (q, *J* = 7.4 Hz, 0.5H), 4.55 (dt, *J* = 5.5, 1.5 Hz, 2H), 4.41 (t, *J* = 9.0 Hz, 1H), 4.36–4.31 (m, 1H), 4.28 (t, *J* = 7.9 Hz, 0.5H), 4.21–4.10 (m, 2H), 3.75 (dd, *J* = 8.8, 7.0 Hz, 0.5H), 3.66 (t, *J* = 8.0 Hz, 0.5H), 3.52–3.43 (m, 1H), 3.41–3.35 (m, 0.5H), 2.94–2.84 (m, 2H), 2.84–2.77 (m, 0.5H), 2.76–2.68 (m, 0.5H), 2.21–2.11 (m, 0.5H), 2.08–1.96 (m, 2H), 1.95–1.87 (m, 1H), 1.81–1.63 (m, 3.5H), 1.59–1.53 (m, 0.5H), 1.36 (s, 4.5H), 1.35 (s, 4.5H), 1.01 (d, *J* = 6.2 Hz, 1.5H), 0.93–0.83 (m, 9H), 0.80 (t, *J* = 6.2 Hz, 6H), 0.77 (d, *J* = 6.8 Hz, 2H), 0.71 (d, *J* = 6.8 Hz, 1.5H), 0.61 (d, *z* = 6.8 Hz, 1H).
^13^C NMR (126 MHz, DMSO-*d*_6_) a mixture of rotamers *δ* 171.9, 171.4, 171.0, 170.9, 170.8, 170.3, 170.0, 169.8, 169.2, 168.5, 168.2, 156.1, 156.1, 155.7, 155.6, 137.4, 136.8, 136.6, 132.3, 129.2, 128.4, 128.4, 128.2, 127.9, 127.9, 127.8, 126.6, 118.1, 118.0, 78.3, 78.2, 70.9, 65.9, 65.8, 64.7, 64.7, 60.0, 59.5, 59.1, 58.1, 57.9, 57.6, 57.5, 57.4, 52.1, 46.6, 37.9, 32.1, 30.4, 30.1, 29.9, 29.7, 28.6, 28.1, 23.9, 21.9, 19.2, 19.1, 18.9, 18.8, 18.7, 18.5, 18.2, 18.2, 18.0, 16.5, 16.2.
HRMS (FAB+) calcd for C_49_H_71_N_6_O_12_ (M+H^+^) 935.5130, found 935.5132.
[α]^25^_D_ = −22.9 (*c* 1.0, MeOH)

benzyl ((3*S*,6*S*,9*S*,12*R*,13*S*,16*R*,21a*S*)-16-benzyl-3,6,9-triisopropyl-12-methyl-1,4,7,10,14,17-hexaoxoicosahydropyrrolo[2,1-l][1]oxa[4,7,10,13,16]pentaazacyclononadecin-13-yl)carbamate (**2a**) To a solution of linear hexadepsipeptide **3** (269 mg, 0.15 mmol) and Pd(PPh_3_)_4_ (17 mg, 0.02 mmol) in dry THF (1.5 mL) was added *N*-methylaniline (0.03 mL, 0.80 mmol) at room temperature. After stirring for 2 h at the same temperature, the reaction mixture was quenched with 1 M HCl and extracted with EtOAc. The combined organic layer was dried over MgSO_4_ and concentrated in vacuo. The residue was purified by column chromatography on a short pad of silica gel (CH_2_Cl_2_/MeOH = 9:1 to 6:1) to afford 256 mg (quant.) of the acid **12** as a yellow solid. To a solution of the above acid **12** (65 mg, 0.07 mmol) in dry CH_2_Cl_2_ (40 mL) was subsequently added TFA (10 mL) at room temperature. After stirring for 20 min at room temperature, the reaction mixture was concentrated in vacuo to afford TFA salt as a white powder. This cyclization precursor was used in the next step without further purification. To a solution of the above cyclization precursor (0.07 mmol) and DIPEA (0.06 mL, 0.37 mmol) in DMF (73 mL) was added DEPBT (109 mg, 0.37 mmol) at room temperature. After stirring for 2 days at the same temperature, the reaction mixture was quenched with 1 M HCl and extracted with EtOAc. The combined organic layer was washed with aqueous NaHCO_3_, dried over MgSO_4_ and concentrated in vacuo. The residue was purified by flash column chromatography (Acetone/Hexane = 1:2 to 2:3) to give 48 mg (85% for 3 steps) of cyclic hexadepsipeptide **2a** as a white powder.

^1^H NMR (500 MHz, DMSO-*d*_6_) *δ* 8.74 (s, 1H), 8.63 (d, *J* = 5.6 Hz, 1H), 8.12 (d, *J* = 9.1 Hz, 1H), 7.42–7.26 (m, 8H), 7.26–7.20 (m, 1H), 7.19–7.14 (m, 2H), 5.08–4.97 (m, 2H), 4.95–4.87 (m, 1H), 4.70 (dt, *J* = 10.0, 5.1 Hz, 1H), 4.54 (dd, *J* = 8.9, 3.6 Hz, 1H), 4.34 (dd, *J* = 8.6, 7.1 Hz, 1H), 3.91 (dd, *J* = 8.5, 3.4 Hz, 1H), 3.72 (dd, *J* = 6.9, 2.1 Hz, 1H), 3.63 (t, *J* = 9.9 Hz, 1H), 3.41–3.34 (m, 1H), 3.26 (dd, *J* = 12.6, 4.4 Hz, 1H), 2.76 (dd, *J* = 12.4, 9.4 Hz, 1H), 2.43–2.35 (m, 1H), 2.15–2.07 (m, 1H), 1.98 (h, *J* = 6.8 Hz, 1H), 1.85–1.76 (m, 1H), 1.71–1.55 (m, 3H), 1.42–1.31 (m, 1H), 1.16 (d, *J* = 6.7 Hz, 3H), 1.05 (d, *J* = 6.7 Hz, 3H), 0.96 (d, *J* = 6.7 Hz, 3H), 0.90 (d, *J* = 6.8 Hz, 3H), 0.87–0.83 (m, 6H), 0.76 (d, *J* = 6.5 Hz, 3H).
^13^C NMR (126 MHz, DMSO-*d*_6_) *δ* 171.7, 171.4, 170.9, 169.7, 168.9, 167.4, 155.3, 137.0, 136.7, 129.2, 128.4, 128.3, 127.8, 127.7, 126.7, 79.2, 69.2, 65.6, 62.3, 60.9, 60.5, 55.6, 54.4, 53.8, 46.6, 32.1, 29.3, 29.2, 28.3, 24.0, 19.7, 19.6, 19.1, 18.8, 18.2, 18.1, 14.1.
HRMS (FAB+) calcd for C_41_H_57_N_6_O_9_ (M+H^+^) 777.4187, found 777.4188.
[α]^25^_D_ = +2.6 (*c* 1.0, MeOH)

*tert*-butyl ((*S*)-1-(((3*S*,6*S*,9*S*,12*R*,13*S*,16*R*,21a*S*)-16-benzyl-3,6,9-triisopropyl-12-methyl-1,4,7,10,14,17-hexaoxoicosahydropyrrolo[2,1-l][1]oxa[4,7,10,13,16]pentaazacyclononadecin-13-yl)amino)-3-methyl-1-oxobutan-2-yl)carbamate (**13a**) To a solution of cyclic hexadepsipeptide **2a** (33 mg, 0.04 mmol) in MeOH (2 mL) was added palladium on activated carbon (15 mg) and the reaction flask was thoroughly sealed. After stirring for 4 h under H_2_ atmosphere, the reaction mixture was filtered through a pad of Celite. The filtered solution was concentrated in vacuo. The corresponding amine was directly used in the next step without further purification. To a solution of the above amine (0.04 mmol), *N*-Boc-l-Val-OH (28 mg, 0.13 mmol) and DIPEA (0.04 mL, 0.25 mmol) in DMF (2 mL) was added HATU (48 mg, 0.13 mmol) at 0 °C. After stirring overnight at room temperature, the reaction mixture was quenched with 1 M HCl and extracted with EtOAc. The combined organic layer was washed with saturated NaHCO_3_, dried over MgSO_4_ and concentrated in vacuo. The residue was purified by flash column chromatography on silica gel (Acetone/Hexane = 1:2 to 2:3) to give 29 mg (82% over 2 steps) of cyclic heptadepsipeptide **13a** as a white powder.

^1^H NMR (500 MHz, DMSO-*d*_6_) *δ* 8.80–8.70 (m, 2H), 8.06 (d, *J* = 9.2 Hz, 1H), 7.68–7.51 (m, 2H), 7.38 (d, *J* = 8.7 Hz, 1H), 7.31–7.26 (m, 2H), 7.24–7.21 (m, 1H), 7.20–7.16 (m, 2H), 6.99 (d, *J* = 8.8 Hz, 1H), 4.94 (dt, *J* = 10.8, 5.4 Hz, 1H), 4.78–4.69 (m, 2H), 4.36 (dd, *J* = 8.6, 6.5 Hz, 1H), 3.93 (dd, *J* = 8.5, 3.5 Hz, 1H), 3.82 (dd, *J* = 8.8, 6.1 Hz, 1H), 3.71 (dd, *J* = 7.0, 2.2 Hz, 1H), 3.63 (t, *J* = 10.0 Hz, 1H), 3.18 (dd, *J* = 12.8, 5.0 Hz, 1H), 2.84 (dd, *J* = 12.7, 8.9 Hz, 1H), 2.57–2.52 (m, 1H), 2.17–2.08 (m, 1H), 2.05–1.92 (m, 2H), 1.91–1.82 (m, 1H),z 1.77–1.69 (m, 1H), 1.68–1.58 (m, 2H), 1.41 (s, 9H), 1.06 (dd, *J* = 9.1, 6.7 Hz, 6H), 0.96 (d, *J* = 6.8 Hz, 3H), 0.90 (d, *J* = 6.7 Hz, 3H), 0.86 (d, *J* = 6.6 Hz, 6H), 0.83 (d, *J* = 6.9 Hz, 3H), 0.81 (d, *J* = 6.7 Hz, 3H), 0.76 (d, *J* = 6.5 Hz, 3H).
^13^C NMR (126 MHz, DMSO-*d*_6_) *δ* 171.8, 171.4, 170.9, 170.3, 169.4, 168.8, 166.9, 155.6, 136.8, 131.5, 131.4, 129.3, 128.8, 128.7, 128.2, 126.6, 78.2, 68.2, 62.4, 61.1, 60.6, 59.8, 55.4, 53.6, 52.2, 46.6, 35.8, 32.2, 30.8, 30.1, 29.3, 29.2, 28.3, 28.2, 24.0, 19.6, 19.6, 19.3, 19.2, 18.9, 18.1, 18.0, 17.9, 13.8.
HRMS (FAB+) calcd for C_43_H_68_N_7_NaO_10_ (M+Na^+^) 864.4847, found 864.4844.
[α]^25^_D_ = +11.2 (*c* 1.0, MeOH)

Xentrivalpeptide A (**1a**) The cyclic heptadepsipeptide **13a** (35 mg, 0.04 mmol) was treated with 4 M HCl in 1,4-dioxane (2 mL) and stired for 1.5 h at room temperature. The reaction mixture was concentrated in vacuo to afford HCl salt as a white powder. The amine was used in the next step without further purification. To a solution of the above amine (0.04 mmol) and DIPEA (0.04 mL, 0.21 mmol) in DMF (2 mL) was added phenylacetyl chloride (0.02 mL, 0.12 mmol) at 0 °C. After stirring overnight at room temperature, the reaction mixture was quenched with 1 M HCl and extracted with EtOAc. The combined organic layer was washed with saturated NaHCO_3_, dried over MgSO_4_ and concentrated in vacuo. The residue was purified by flash column chromatography on silica gel (CHCl_3_/MeOH = 19:1) to give 26 mg (73% over 2 steps) of xentrivalpeptide A (**1a**) as a white powder.

^1^H NMR (500 MHz, CD_3_OD) See Table S1.
^13^C NMR (126 MHz, CD_3_OD) See Table S1.
HRMS (FAB+) calcd for C_46_H_66_N_7_O_9_ (M+H^+^) 860.4922, found 860.4921.
[α]^25^_D_ = −17.1 (*c* 0.36, CHCl_3_)
Melting point 94–96 °C

benzyl ((3*S*,6*R*,9*S*,12*R*,13*S*,16*R*,21a*S*)-16-benzyl-3,6,9-triisopropyl-12-methyl-1,4,7,10,14,17-hexaoxoicosahydropyrrolo[2,1-l][1]oxa[4,7,10,13,16]pentaazacyclononadecin-13-yl)carbamate (**2b**) To a solution of the above acid **12** (319 mg, 0.36 mmol) in dry CH_2_Cl_2_ (40 mL) was subsequently added TFA (10 mL) at room temperature. After stirring for 20 min at room temperature, the reaction mixture was concentrated in vacuo to afford TFA salt as a white powder. This cyclization precursor was used in the next step without further purification. To a solution of PyBOP (926 mg, 1.78 mmol) and DIPEA (0.31 mL, 1.78 mmol) in DMF (130 mL) was added a solution of the cyclization precursor (0.36 mmol) in DMF (50 mL) via syringe pump over a period of 10 h at room temperature. After stirring for 2 days at the same temperature, the reaction mixture was quenched with 1 M HCl and extracted with EtOAc. The combined organic layer was washed with aqueous NaHCO_3_, dried over MgSO_4_ and concentrated in vacuo. The residue was purified by flash column chromatography (EtOAc/Hexane = 1:1 to 2:1) to give 103 mg (37% over 3 steps) of epimerized cyclic hexadepsipeptide **2b** as a white powder, along with 115 mg of **2a**.

^1^H NMR (500 MHz, DMSO-*d*_6_) *δ* 8.65 (s, 1H), 7.99 (d, *J* = 9.4 Hz, 1H), 7.40–7.26 (m, 8H), 7.22 (t, *J* = 7.8 Hz, 2H), 7.19–7.13 (m, 2H), 6.76 (s, 1H), 5.03 (s, 2H), 5.02–4.96 (m, 1H), 4.85 –4.77 (m, 1H), 4.26–4.21 (m, 2H), 4.20–4.16 (m, 1H), 4.03 (dd, *J* = 7.2, 3.0 Hz, 1H), 3.96 (dd, *J* = 9.4, 6.9 Hz, 1H), 3.66–3.59 (m, 1H), 3.48–3.40 (m, 1H), 3.00–2.95 (m, 2H), 2.21–2.11 (m, 2H), 2.09–2.01 (m, 1H), 2.00–1.92 (m, 1H), 1.82–1.65 (m, 3H), 0.93 (d, *J* = 6.6 Hz, 3H), 0.90 (dd, *J* = 6.8, 4.2 Hz, 6H), 0.86 (d, *J* = 6.8 Hz, 3H), 0.79 (d, *J* = 6.7 Hz, 6H), 0.69 (d, *J* = 6.8 Hz, 3H).
^13^C NMR (126 MHz, DMSO-*d*_6_) *δ* 171.9, 171.7, 171.1, 170.6, 170.1, 167.4, 155.9, 137.7, 136.8, 129.7, 128.3, 127.9, 127.9, 127.6, 126.1, 79.2, 69.4, 65.7, 61.0, 59.7, 57.2, 57.1, 51.4, 46.9, 46.6, 46.6, 36.2, 30.2, 29.3, 29.2, 28.8, 24.5, 19.2, 19.1, 18.8, 17.7, 17.1, 14.6.
HRMS (FAB+) calcd for C_41_H_57_N_6_O_9_ (M+H^+^) 777.4187, found 777.4184.
[α]^25^_D_ = +8.0 (*c* 1.0, MeOH)

*tert*-butyl ((*S*)-1-(((3*S*,6*R*,9*S*,12*R*,13*S*,16*R*,21a*S*)-16-benzyl-3,6,9-triisopropyl-12-methyl-1,4,7,10,14,17-hexaoxoicosahydropyrrolo[2,1-l][1]oxa[4,7,10,13,16]pentaazacyclononadecin-13-yl)amino)-3-methyl-1-oxobutan-2-yl)carbamate (**13b**) To a solution of cyclic hexadepsipeptide **2b** (39 mg, 0.05 mmol) in MeOH (2 mL) was added palladium on activated carbon (15 mg) and the reaction flask was thoroughly sealed. After stirring for 2.5 h under H_2_ atmosphere, the reaction mixture was filtered through a pad of Celite. The filtered solution was concentrated in vacuo. The corresponding amine was directly used in the next step without further purification. To a solution of the above amine (0.05 mmol), *N*-Boc-l-Val-OH (33 mg, 0.15 mmol) and DIPEA (0.05 mL, 0.30 mmol) in DMF (2 mL) was added HATU (57 mg, 0.15 mmol) at 0 °C. After stirring overnight at room temperature, the reaction mixture was quenched with 1 M HCl and extracted with EtOAc. The combined organic layer was washed with saturated NaHCO_3_, dried over MgSO_4_ and concentrated in vacuo. The residue was purified by flash column chromatography on silica gel (Acetone/Hexane = 1:2) to give 36 mg (86% over 2 steps) of cyclic heptadepsipeptide **13b** as a white powder.

^1^H NMR (500 MHz, DMSO-*d*_6_) *δ* 8.47 (s, 1H), 7.80 (d, *J* = 7.2 Hz, 1H), 7.55 (s, 1H), 7.29 (d, *J* = 7.0 Hz, 2H), 7.23 (t, *J* = 7.4 Hz, 2H), 7.20–7.14 (m, 2H), 6.75 (d, *J* = 9.1 Hz, 1H), 5.03 (q, *J* = 8.5 Hz, 1H), 4.89 (d, *J* = 6.8 Hz, 1H), 4.36–4.15 (m, 4H), 3.97 (dd, *J* = 9.7, 7.5 Hz, 1H), 3.83 (dd, *J* = 9.1, 6.3 Hz, 1H), 3.57–3.41 (m, 2H), 3.02–2.89 (m, 2H), 2.24–2.14 (m, 1H), 2.14–2.04 (m, 2H), 2.02–1.95 (m, 1H), 1.94–1.86 (m, 1H), 1.85–1.79 (m, 1H), 1.79–1.67 (m, 2H), 1.37 (s, 9H), 0.94–0.88 (m, 9H), 0.87–0.84 (m, 9H), 0.81 (d, *J* = 6.8 Hz, 3H), 0.78–0.72 (m, 6H).
^13^C NMR (126 MHz, DMSO-*d*_6_) *δ* 172.3, 171.2, 171.1, 171.0, 170.5, 170.1, 167.2, 155.6, 137.7, 129.6, 127.9, 126.2, 78.3, 69.0, 59.5, 59.2, 57.4, 54.8, 51.6, 47.0, 38.2, 36.2, 30.4, 30.3, 29.2, 29.2, 29.0, 28.1, 24.6, 19.3, 19.2, 19.0, 18.9, 17.8, 17.4, 14.6.
HRMS (FAB+) calcd for C_43_H_68_N_7_O_10_ (M+H^+^) 842.5028, found 842.5031.
[α]^25^_D_ = +2.6 (*c* 0.1, MeOH)

6α-*epi*-Xentrivalpeptide A (**1b**) The cyclic heptadepsipeptide **13b** (20 mg, 0.02 mmol) was treated with 4 M HCl in 1,4-dioxane (1 mL) and stired for 2 h at room temperature. The reaction mixture was concentrated in vacuo to afford HCl salt as a white powder. The amine was used in the next step without further purification. To a solution of the above amine (0.02 mmol) and DIPEA (0.01 mL, 0.07 mmol) in DMF (1 mL) was added phenylacetyl chloride (0.01 mL, 0.07 mmol) at 0 °C. After stirring overnight at room temperature, the reaction mixture was quenched with 1 M HCl and extracted with EtOAc. The combined organic layer was washed with saturated NaHCO_3_, dried over MgSO_4_ and concentrated in vacuo. The residue was purified by flash column chromatography on silica gel (Acetone/Hexane = 2:3) to give 12 mg (58% over 2 steps) of 6α-*epi*-xentrivalpeptide A (**1b**) as a white powder.

^1^H NMR (500 MHz, CD_3_OD) See Table S2.
^13^C NMR (126 MHz, CD_3_OD) See Table S2.
HRMS (FAB+) calcd for C_46_H_66_N_7_O_9_ (M+H^+^) 860.4922, found 860.4917.
[α]^25^_D_ = +5.1 (*c* 0.36, CHCl_3_)
Melting point 134–136 °C

## 4. Conclusions

In summary, the first total synthesis of xentrivalpeptide A was achieved in 15 steps in the longest linear sequence with 33% overall yield using a solution-phase condensation strategy. Notably, switching the coupling reagent from DEPBT to PyBOP in the macrolactamization step enabled access to 6α-*epi*-xentrivalpeptide A. The modular nature of this synthetic strategy should facilitate its extension to the preparation of other members of the xentrivalpeptide family. Further studies exploring the biological activities of this natural product are ongoing.

## Figures and Tables

**Table 1:** Screening of macrolactamization conditions.

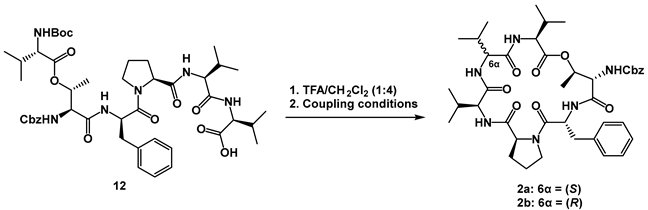			
Entry	Conditions ^a^	Cyclization Efficiency ^b^	d.r. (2a:2b)
1	EDC, DIPEA, HOAt, CH_2_Cl_2_	0.23	**2a** only
2	HATU, DIPEA, CH_2_Cl_2_	0.21	**2a** only
3	PyBOP, DIPEA, CH_2_Cl_2_	0.19	3.2:1
4	DEPBT, DIPEA, CH_2_Cl_2_	0.23	**2a** only
5	EDC, DIPEA, HOAt, DMF	0.19	2.7:1
6	HATU, DIPEA, DMF	0.12	3:1
7	PyBOP, DIPEA, DMF	0.15	1:1
8	DEPBT, DIPEA, DMF	0.31	**2a** only
9	PyBOP, DIPEA, DMF, slow addition	0.18 ^c^	1:1.8

^a^ All reactions were carried out using 5 equiv. of coupling reagent and 5 equiv. of DIPEA at a substrate concentration of 1 mM. HOAt (5 equiv.) was additionally used in entries 1 and 5. ^b^ Determined from the HPLC peak area ratio of **2a** to the internal standard. ^c^ HPLC peak area ratio of **2b** to the internal standard.

## Data Availability

Data are contained within the article and Supporting Information.

## Supplementary Materials

The following supporting information can be downloaded at https://www.mdpi.com/article/10.3390/molecules31183340/s1, which includes an NMR assignment table comparing natural and synthetic xentrivalpeptide A, detailed optimization procedures and HPLC traces for the macrolactamization, and copies of the ^1^H and ^13^C NMR spectra; Table S1: Comparison of 1H and 13C NMR spectral data of natural and synthetic xentrivalpeptide A (**1a**); Table S2: Comparison of 1H and 13C NMR spectral data of 6α-*epi* xentrivalpeptide A (**1b**) and xentrivalpeptide A (**1a**); Figure S1: Structure of the internal standard used for the HPLC analysis.

## Abbreviations

The following abbreviations are used in this manuscript:

DEPBT	3-(diethoxyphosphoryloxy)-1,2,3-benzotriazin-4(3H)-one
PyBOP	benzotriazol-1-yloxytripyrrolidinophosphonium hexafluorophosphate
TFA	Trifluoroacetic acid
HATU	*O*-(7-azabenzotriazol-1-yl)-*N*,*N*,*N*′,*N*′-tetramethyluronium hexafluorophosphate
DIPEA	*N*,*N*-Diisopropylethylamine
EDC	1-Ethyl-3-(3-dimethylaminopropyl)carbodiimide
DMAP	4-Dimethylaminopyridine
THF	Tetrahydrofuran
HOAt	1-Hydroxy-7-azabenzotriazole
DMF	*N*,*N*-Dimethylformamide
