# Supplementary material for: Total Synthesis of Xentrivalpeptide A and Its 6α-epi Derivative
Source: Molecules. 2026 Sep 20;31(18):3340. doi: 10.3390/molecules31183340 (PMC13609800; doi:10.3390/molecules31183340)
Supplement: Supplementary file 1 [file molecules-31-03340-s001.zip › molecules-4565569-supplementary.pdf]

Supporting Information

Total Synthesis of Xentrivalpeptide A and its 6-*epi* Derivative

Joonseong Hur,[1,*] Yunseong Lee[1]

[1] College of Pharmacy and Inje Institute of Pharmaceutical Sciences and Research, Inje University, 197 Inje-ro, Gimhae-si, Gyeongnam 50834, Republic of Korea

Table of contents

NMR Assignment of xentrivalpeptide A **S3**

Optimization of macrolactamization **S6**

Copies of NMR Spectra **S13**

References **S28**

NMR Assignment of xentrivalpeptide A

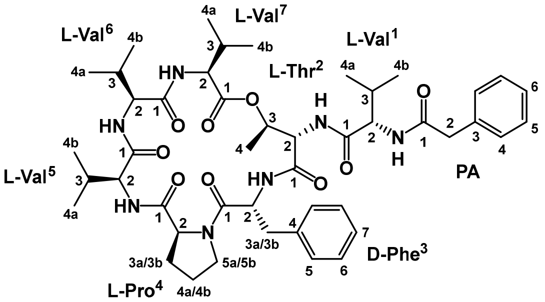

Xentrivalpeptide A (1a)

Table S1. Comparison of ^{1}H and ^{13}C NMR spectral data of natural and synthetic xentrivalpeptide A (**1a**)

Subunit	Position	Natural xentrivalpeptide A (500 MHz)[1]		Synthetic xentrivalpeptide A (**1a**; 500 MHz)		Δ*δ*, ppm (Natural – synthetic **1a**)	
		^{1}H (*δ*, mult, *J* in Hz)	^{13}C	^{1}H (*δ*, mult, *J* in Hz)	^{13}C	ΔH	ΔC
PA	1	-	174.2	-	174.2	-	0
	2	3.60, d, (1.0)	43.6	3.59, d, (1.0)	43.7	0.01	-0.1
	3	-	137.0	-	137.0	-	0
	4	7.29, m	130.3	7.28, m, overlapped	130.3	0.01	0
	5	7.30, m	129.7	7.28, m, overlapped	129.7	0.02	0
	6	7.25, m	128.2	7.22, m, overlapped	128.3	0.03	-0.1
L-Val^{1}	1	-	172.8	-	172.8	-	0
	2	4.25, d, (7.3)	60.2	4.25, d, (7.4)	60.3		-0.1
	3	2.10, m	31.7	2.08, m, overlapped	31.9	0.02	-0.2
	4a	0.94, d, (6.7)	19.8	0.94, d, (6.8)	19.9	0	-0.1
	4b	0.92, d, (6.8)	18.6	0.92, d, (5.2)	18.7	0	-0.1
L-Thr^{2}	1	-	169.5	-	169.5	-	0
	2	4.79, d, (3.8)	55.0	4.79, d, (3.7)	55.0	0	0
	3	5.09, m	69.9	5.08, m	70.1	0.01	-0.2
	4	1.14, d, (2.4)	14.6	1.13, d, (7.0)	14.6	0.01	0
D-Phe^{3}	1	-	171.3	-	171.3	-	0
	2	4.94, dd, (9.9, 5.2)	55.3	4.93, dd, (9.9, 5.0)	55.3	0.01	0
	3a	2.91, dd, (12.6, 9.8)	40.5	2.91, dd, (12.6, 10.0)	40.6	0	-0.1
	3b	3.24, dd, (12.5, 5.0)	40.5	3.24, dd, (12.6, 5.0)	40.6	0	-0.1
	4	-	137.0	-	137.0	-	0
	5	7.29−7.27	130.6	7.28, m, overlapped	130.7	0	-0.1
	6	7.29−7.27	129.7	7.28, m, overlapped	129.7	0	0
	7	7.24−7.20	127.9	7.22, m, overlapped	127.9	0	0
L-Pro^{4}	1	-	174.9	-	175.0	-	-0.1
	2	4.09, dd, (8.7, 4.9)	62.4	4.09, dd, (8.4, 4.5)	62.7	0	-0.3
	3a	1.79, m	30.7	1.78, m, overlapped	30.7	0.01	0
	3b	1.89, m	30.7	1.88, m	30.7	0.01	0
	4a	1.47, m	25.5	1.47, m	25.7	0	-0.2

Subunit	Position	Natural xentrivalpeptide A (500 MHz)[1]		Synthetic xentrivalpeptide A (**1a**; 500 MHz)		Δδ, ppm (Natural – synthetic **1a**)	
		^{1}H (δ, mult, J in Hz)	^{13}C	^{1}H (δ, mult, J in Hz)	^{13}C	ΔH	ΔC
	4b	1.79, m	25.5	1.78, m, overlapped	25.7	0.01	-0.2
	5a	2.68, m	48.6	2.68, m	48.7	0	-0.1
	5b	3.44, m	48.6	3.44, m	48.7	0	-0.1
L-Val^{5}	1	-	173.6	-	173.7	-	-0.1
	2	3.64, d (11.3)	65.5	3.64, d (10.8)	65.7	0	-0.2
	3	2.31, m	30.8	2.30, m	30.8	0.01	0
	4a	0.89, d (6.4)	20.3	0.89, d (6.5)	20.4	0	-0.1
	4b	0.98, d (6.4)	20.1	0.97, d (6.7)	20.1	0.01	0
L-Val^{6}	1	-	173.5	-	173.5	-	0
	2	4.42, d (5.9)	58.0	4.41, d (6.2)	58.0	0.01	0
	3	2.04, m	33.6	2.06, m, overlapped	33.8	-0.02	-0.2
	4a	0.93, d (7.0)	18.7	0.93, d (5.3)	18.7	0	0
	4b	0.99, d (7.0)	19.7	0.98, d (6.7)	19.8	0.01	-0.1
L-Val^{7}	1	-	172.0	-	172.0	-	0
	2	3.94, d (7.7)	62.4	3.94, d (7.5)	62.6	0	-0.2
	3	2.07, m	30.2	2.06, m, overlapped	30.3	0.01	-0.1
	4a	1.13, d (6.7)	19.8	1.13, d (6.8)	19.9	0	-0.1
	4b	1.04, d (6.8)	18.9	1.03, d (6.8)	18.9	0.01	0

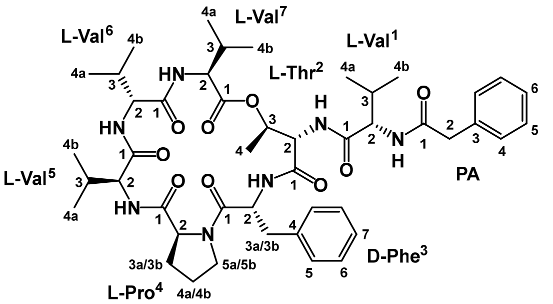

6α-*epi* xentrivalpeptide A (1b)

Table S2. Comparison of ^{1}H and ^{13}C NMR spectral data of 6α-*epi* xentrivalpeptide A (**1b**) and xentrivalpeptide A (**1a**)

Subunit	Position	6α-*epi* xentrivalpeptide A (**1b**; 500 MHz)		xentrivalpeptide A (**1a**; 500 MHz)		Δδ, ppm (**1b** –**1a**)	
		^{1}H (δ, mult, J in Hz)	^{13}C	^{1}H (δ, mult, J in Hz)	^{13}C	ΔH	ΔC
PA	1	-	173.8	-	174.2	-	-0.4
	2a	3.65, d, (14.0)	43.7	3.59, d, (1.0)	43.7	0[a]	0
	2b	3.54, d, (14.0)					
	3	-	137.0	-	137.0	-	0
	4	7.28, m, overlapped	130.3	7.28, m, overlapped	130.3	0	0
	5	7.28, m, overlapped	129.8	7.28, m, overlapped	129.7	0	0.1
	6	7.22, m, overlapped	128.2	7.22, m, overlapped	128.3	0	-0.1

Subunit	Position	6α-*epi* xentrivalpeptide A (**1b**; 500 MHz)		xentrivalpeptide A (**1a**; 500 MHz)		Δ*δ*, ppm (**1b** –**1a**)	
		^{1}H (*δ*, mult, *J* in Hz)	^{13}C	^{1}H (*δ*, mult, *J* in Hz)	^{13}C	ΔH	ΔC
L-Val^{1}	1	-	174.1	-	172.8	-	1.3
	2	4.08, d, (7.8)	61.5	4.25, d, (7.4)	60.3	-0.17	1.2
	3	2.24, m, overlapped	31.5	2.08, m, overlapped	31.9	0.16	-0.4
	4a	1.15, d, (6.7)	20.5	0.94, d, (6.8)	19.9	0.21	0.6
	4b	1.03, d, (6.6)	19.4	0.92, d, (5.2)	18.7	0.11	0.7
L-Thr^{2}	1	-	171.1	-	169.5	-	1.6
	2	4.77, d, (1.6)	56.2	4.79, d, (3.7)	55.0	-0.02	1.2
	3	5.40, q, (5.3)	71.0	5.08, m	70.1	0.32	0.9
	4	1.03, d, (6.6)	17.0	1.13, d, (7.0)	14.6	-0.10	2.4
D-Phe^{3}	1	-	172.8	-	171.3	-	1.5
	2	4.97, dd, (9.6, 6.2)	54.7	4.93, dd, (9.9, 5.0)	55.3	0.04	-0.6
	3a	2.88, dd, (13.2, 6.1)	38.3	2.91, dd, (12.6, 10.0)	40.6	-0.03	-2.3
	3b	3.00, dd, (13.1, 9.6)	38.3	3.24, dd, (12.6, 5.0)	40.6	-0.24	-2.3
	4	-	138.1	-	137.0	-	1.1
	5	7.28, m, overlapped	130.5	7.28, m, overlapped	130.7	0	-0.2
	6	7.28, m, overlapped	129.8	7.28, m, overlapped	129.7	0	0.1
	7	7.22, m, overlapped	128.2	7.22, m, overlapped	127.9	0	0.3
L-Pro^{4}	1	-	175.4	-	175.0	-	0.4
	2	4.38, t, (8.1)	60.8	4.09, dd, (8.4, 4.5)	62.7	0.29	-1.9
	3a	1.60, m, overlapped	30.4	1.78, m, overlapped	30.7	-0.18	-0.3
	3b	2.27, m, overlapped	30.4	1.88, m	30.7	0.39	-0.3
	4a	1.58, m, overlapped	26.1	1.47, m	25.7	0.11	0.4
	4b	1.76, m, overlapped	26.1	1.78, m, overlapped	25.7	-0.02	0.4
	5a	3.12, m	49.1	2.68, m	48.7	0.44	0.4
	5b	3.27, m	49.1	3.44, m	48.7	-0.17	0.4
L-Val^{5}	1	-	175.9	-	173.7	-	2.2
	2	4.26, d, (9.4)	62.4	3.64, d (10.8)	65.7	0.62	-3.3
	3	1.87, m	30.6	2.30, m	30.8	-0.43	-0.2
	4a	0.78, d, (7.1)	20.0	0.89, d (6.5)	20.4	-0.11	-0.4
	4b	0.98, d, (6.6)	20.7	0.97, d (6.7)	20.1	0.01	0.6
D-Val^{6}	1	-	172.9	-	173.5	-	-0.6
	2	4.15, m	60.6	4.41, d (6.2)	58.0	-0.26	2.6
	3	2.30, m, overlapped	30.8	2.06, m, overlapped	33.8	0.24	-3.0
	4a	0.77, d, (7.0)	17.1	0.93, d (5.3)	18.7	-0.16	-1.6
	4b	0.91, d, (7.0)	20.2	0.98, d (6.7)	19.8	-0.07	0.4
L-Val^{7}	1	-	170.2	-	172.0	-	-1.8
	2	4.10, d, (9.5)	60.8	3.94, d (7.5)	62.6	0.16	-1.8
	3	1.71, m, overlapped	32.2	2.06, m, overlapped	30.3	-0.35	1.9
	4a	0.94, d, (6.7)	19.4	1.13, d (6.8)	19.9	-0.19	-0.5
	4b	0.80, d, (7.0)	20.4	1.03, d (6.8)	18.9	-0.23	1.5

[a] The average of the two separated proton signals in the spectrum of **1b** was used for comparison.

Optimization of macrolactamization

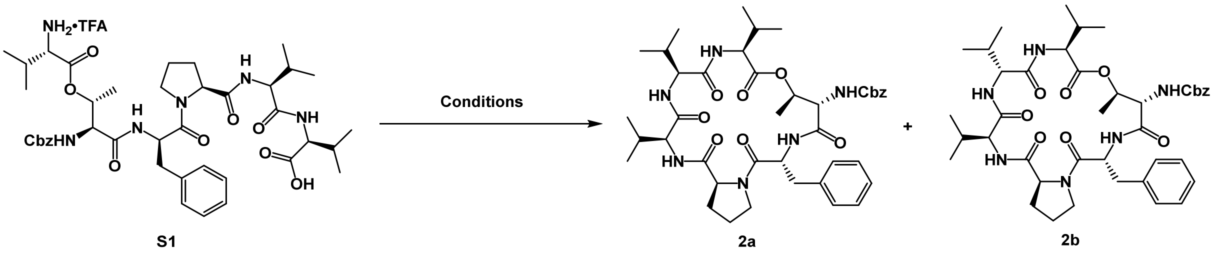

The internal standard solution was prepared by dissolving 2 mg of tripeptide **S2** (Figure S1) in HPLC-grade acetonitrile and diluting to a final volume of 20 mL. The HPLC peak of the internal standard **S2** was well separated from those of products **2a** and **2b** without any overlap.

To a solution of the deprotected cyclization precursor **S1** (10 mg, 0.01 mmol) and DIPEA (0.01 mL, 0.06 mmol, 5 equiv.) in the indicated solvent (10 mL, 1 mM) was added to the coupling reagent (0.06 mmol, 5 equiv.) at room temperature. For entries 1 and 5, HOAt (7 mg, 0.06 mmol, 5 equiv.) was added prior to the addition of the coupling reagent. After stirring overnight at room temperature, a 100 μL aliquot of the reaction mixture was withdrawn, transferred to a 1/2-dram vial, and concentrated to dryness under reduced pressure. The resulting residue was dissolved in 1.0 mL of the internal standard solution and analyzed by HPLC. The relative formation of **2a** was evaluated based on the ratio of the HPLC peak area of **2a** to that of the internal standard **S2**.

HPLC analysis was performed using eluent A (0.1% formic acid in HPLC-grade acetonitrile) and eluent B (0.1% formic acid in double-distilled water) at a flow rate of 1.0 mL/min. Diastereomeric ratio was determined by integration of the HPLC peaks of the corresponding reaction mixture at a wavelength of 210 nm. The gradient was as follows: 30–95% A over 20 min, 95% A for 5 min, 95–30% A over 0.5 min, and 30% A for 5 min.

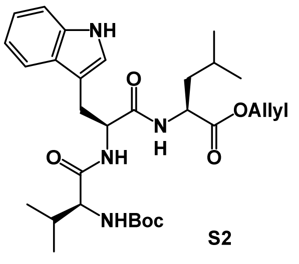

Figure S1. Structure of the internal standard used for the HPLC analysis

Standard

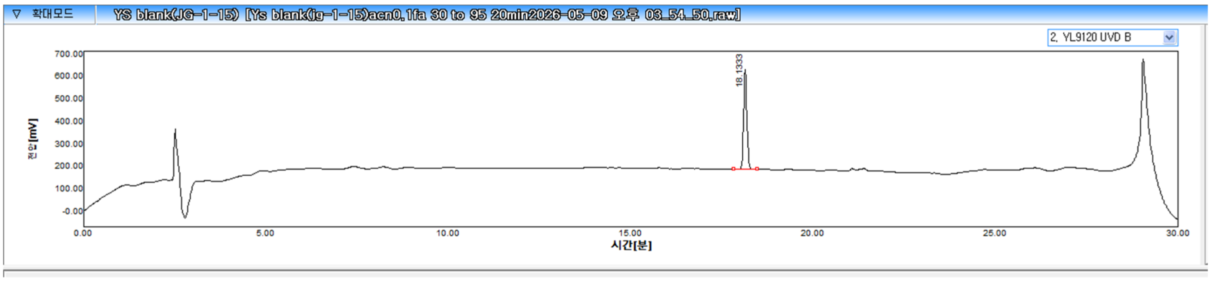

Compound	Retention time (min)	Area (%)	Ratio
**S2**	18.13	100	-

Entry 1. EDC (5.0 eq.), DIPEA (5.0 eq.), HOAt (5.0 eq.), CH_2Cl_2 (1 mM)

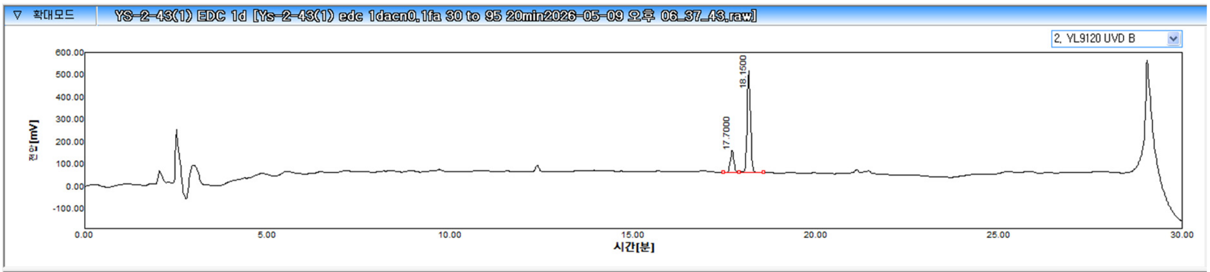

#	피크이름	RT[분]	면적[mV*sec]	면적비[%]	높이[mV]	높이비[%]	농도	농도비[%]	형태	폭[초]	반치폭[초]	이론단수	분리도	Tailing Factor	사용자 정의1	사용자 정의2
1		17,7000	680,0776	18,91	100,0191	18,02	0,000000	0,00	BV	8,66	6,28	240659,0	0,0000	1,0218	0,000000	0,000000
2		18,1500	2917,1567	81,09	455,1477	81,98	0,000000	0,00	VB	9,17	5,88	225513,7	3,0282	1,0655	0,000000	0,000000
합계			3597,2344		555,1668		0,000000									

Compound	Retention time (min)	Area (%)	Ratio
**S2**	18.15	81.09	1.00
**2a**	17.70	18.91	0.23
**2b**	-	-	-

Entry 2. HATU (5.0 eq.), DIPEA (5.0 eq.), CH_2Cl_2 (1 mM)

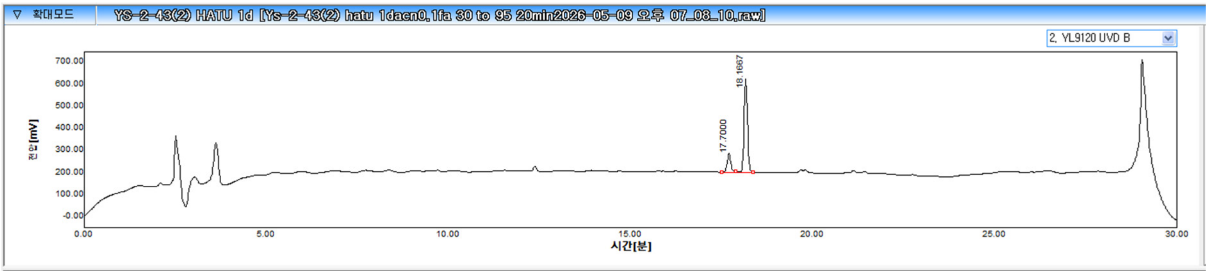

#	피크이름	RT[분]	면적[mV*sec]	면적비[%]	높이[mV]	높이비[%]	농도	농도비[%]	형태	폭[초]	반치폭[초]	이론단수	분리도	Tailing Factor	사용자 정의1	사용자 정의2
1		17.7000	570.9925	17.48	84.5508	16.62	0.000000	0.00	BV	9.70	6.28	191864.0	0.0000	1.0368	0.000000	0.000000
2		18.1667	2696.2166	82.52	424.1522	83.38	0.000000	0.00	VB	7.05	5.90	382619.8	3.3439	0.9787	0.000000	0.000000
합계			3267.2090		508.7029		0.000000									

Compound	Retention time (min)	Area (%)	Ratio
**S2**	18.17	82.52	1.00
**2a**	17.70	17.48	0.21
**2b**	-	-	-

Entry 3. PyBOP (5.0 eq.), DIPEA (5.0 eq.), CH_2Cl_2 (1 mM)

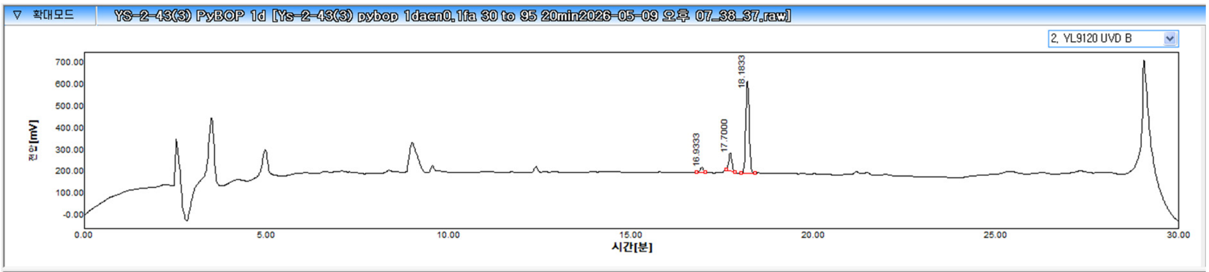

#	피크이름	RT[분]	면적[mV*sec]	면적비[%]	높이[mV]	높이비[%]	농도	농도비[%]	형태	폭[초]	반치폭[초]	이론단수	분리도	Tailing Factor	사용자 정의1	사용자 정의2
1		16.9333	148.8955	4.51	23.1354	4.42	0.000000	0.00	PB	7.06	6.18	331645.2	0.0000	0.8963	0.000000	0.000000
2		17.7000	497.9295	15.07	82.2499	15.71	0.000000	0.00	BB	7.05	5.87	363519.5	6.5236	1.2130	0.000000	0.000000
3		18.1833	2656.8525	80.42	418.0157	79.87	0.000000	0.00	BP	7.05	5.93	383209.8	4.1148	0.9890	0.000000	0.000000
합계			3303.6775		523.4010		0.000000									

Compound	Retention time (min)	Area (%)	Ratio
**S2**	18.18	80.42	1.00
**2a**	17.70	15.07	0.19
**2b**	16.93	4.51	0.06

Entry 4. DEPBT (5.0 eq.), DIPEA (5.0 eq.), CH_2Cl_2 (1 mM)

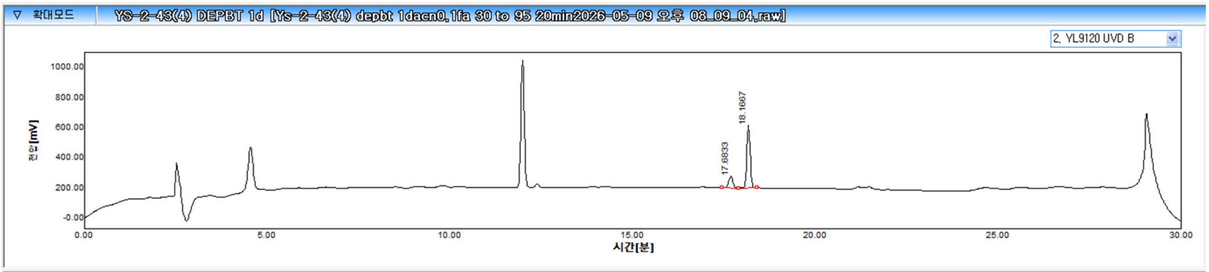

#	피크이름	RT[분]	면적[mV*sec]	면적비[%]	높이[mV]	높이비[%]	농도	농도비[%]	형태	폭[초]	반치폭[초]	이론단수	분리도	Tailing Factor	사용자 정의1	사용자 정의2
1		17.6833	611.6884	18.80	78.8628	16.00	0.000000	0.00	PP	9.31	7.21	207765.6	0.0000	0.9383	0.000000	0.000000
2		18.1667	2642.3276	81.20	414.1681	84.00	0.000000	0.00	PB	7.05	5.93	382498.0	3.5451	0.9871	0.000000	0.000000
합계			3254.0161		493.0309		0.000000									

Compound	Retention time (min)	Area (%)	Ratio
**S2**	18.17	81.20	1.00
**2a**	17.68	18.80	0.23
**2b**	-	-	-

Entry 5. EDC (5.0 eq.), DIPEA (5.0 eq.), HOAt (5.0 eq.), DMF (1 mM)

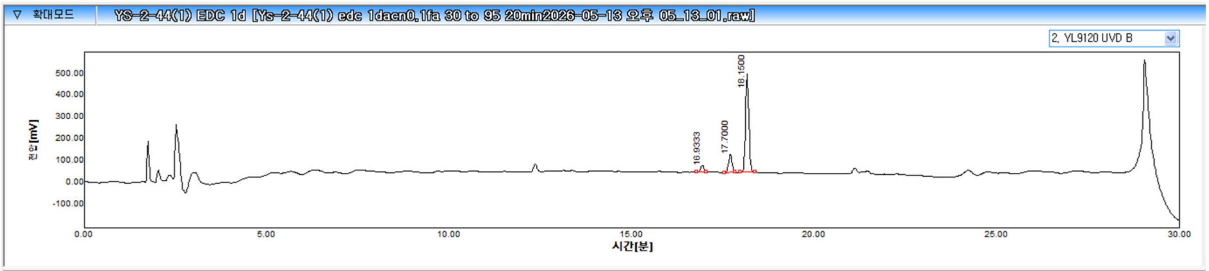

#	피크이름	RT[분]	면적[mV*sec]	면적비[%]	높이[mV]	높이비[%]	농도	농도비[%]	형태	폭[초]	반치폭[초]	이론단수	분리도	Tailing Factor	사용자 정의1	사용자 정의2
1		16.9333	187.7606	5.30	29.7692	5.30	0.000000	0.00	BB	7.05	5.98	331919.7	0.0000	0.8869	0.000000	0.000000
2		17.7000	533.5594	15.07	81.9926	14.60	0.000000	0.00	PB	7.06	6.16	362414.4	6.5200	0.9375	0.000000	0.000000
3		18.1500	2819.3623	79.63	449.8589	80.10	0.000000	0.00	BB	7.05	5.85	381701.5	3.8279	1.0960	0.000000	0.000000
합계			3540.6824		561.6206		0.000000									

Compound	Retention time (min)	Area (%)	Ratio
**S2**	18.15	79.63	1.00
**2a**	17.70	15.07	0.19
**2b**	16.93	5.30	0.07

Entry 6. HATU (5.0 eq.), DIPEA (5.0 eq.), DMF (1 mM)

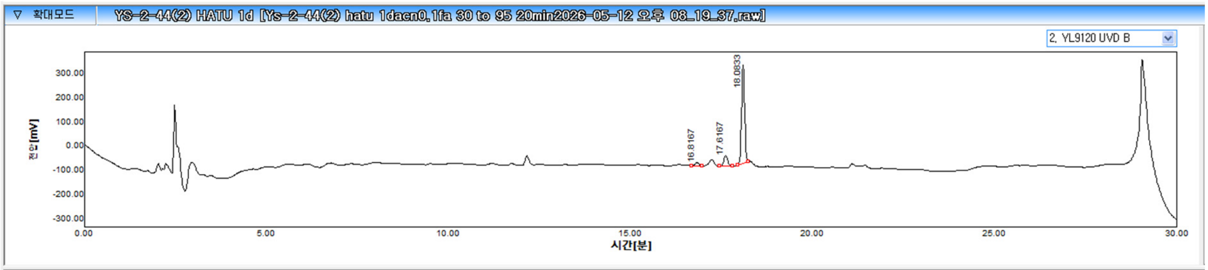

#	피크이름	RT[분]	면적[mV*sec]	면적비[%]	높이[mV]	높이비[%]	농도	농도비[%]	형태	폭[초]	반치폭[초]	이론단수	분리도	Tailing Factor	사용자 정의1	사용자 정의2
1		16,8167	93,1716	3,20	13,1080	2,82	0,000000	0,00	PP	8,46	6,65	227494,9	0,0000	0,9859	0,000000	0,000000
2		17,6167	304,4263	10,45	44,2397	9,53	0,000000	0,00	PP	8,53	6,42	245576,8	5,6492	0,9786	0,000000	0,000000
3		18,0833	2514,5615	86,35	406,9809	87,65	0,000000	0,00	PP	8,41	5,83	266318,3	3,3055	1,0447	0,000000	0,000000
합계			2912,1594		464,3286		0,000000									

Compound	Retention time (min)	Area (%)	Ratio
**S2**	18.08	86.35	1.00
**2a**	17.62	10.45	0.12
**2b**	16.82	3.20	0.04

Entry 7. PyBOP (5.0 eq.), DIPEA (5.0 eq.), DMF (1 mM)

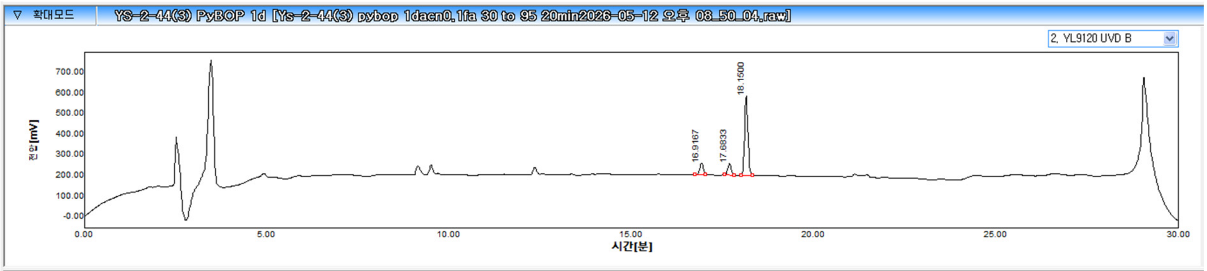

#	피크이름	RT[분]	면적[mV*sec]	면적비[%]	높이[mV]	높이비[%]	농도	농도비[%]	형태	폭[초]	반치폭[초]	이론단수	분리도	Tailing Factor	사용자 정의1	사용자 정의2
1		16,9167	355,0378	11,24	55,3143	11,12	0,000000	0,00	PB	7,84	6,09	268262,8	0,0000	0,9912	0,000000	0,000000
2		17,6833	366,0334	11,59	57,3749	11,53	0,000000	0,00	PB	8,66	6,09	240090,6	5,5757	1,0675	0,000000	0,000000
3		18,1500	2437,6421	77,17	384,7913	77,35	0,000000	0,00	BB	7,05	5,93	381702,1	3,5642	1,0072	0,000000	0,000000
합계			3158,7134		497,4805		0,000000									

Compound	Retention time (min)	Area (%)	Ratio
**S2**	18.15	77.17	1.00
**2a**	17.68	11.59	0.15
**2b**	16.92	11.24	0.15

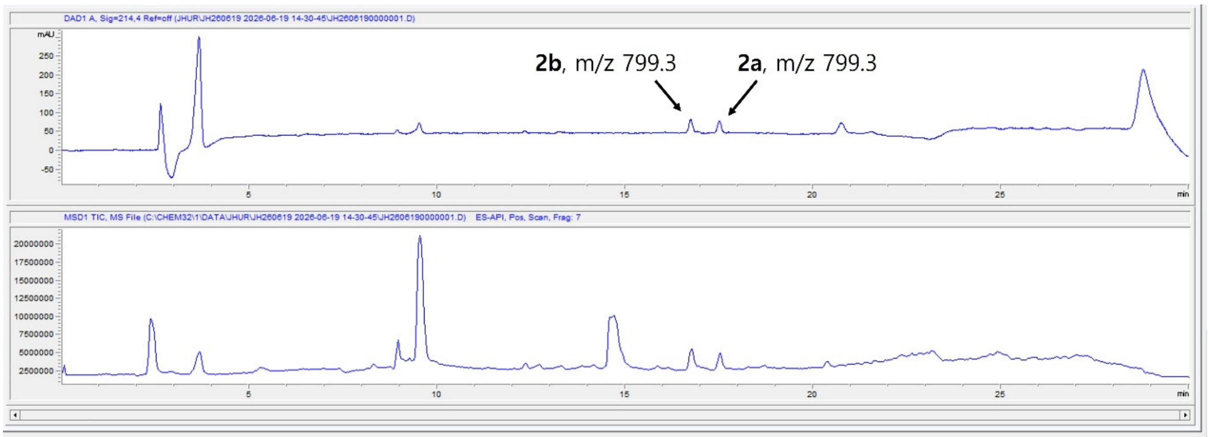

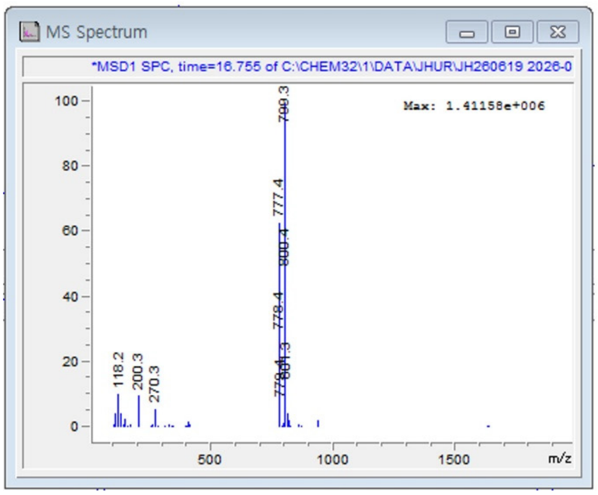

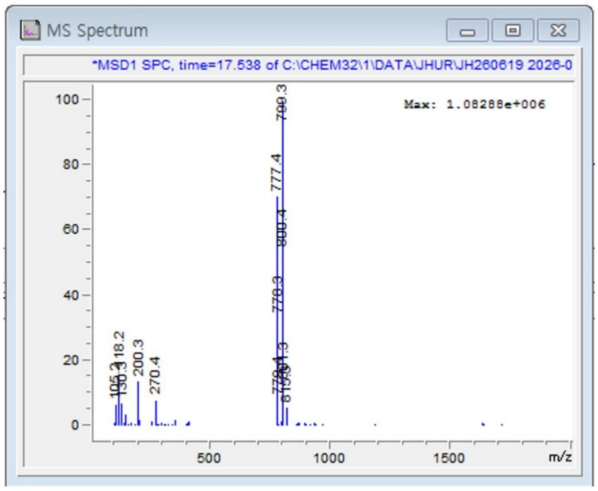

Entry 8. DEPBT (5.0 eq.), DIPEA (5.0 eq.), DMF (1 mM)

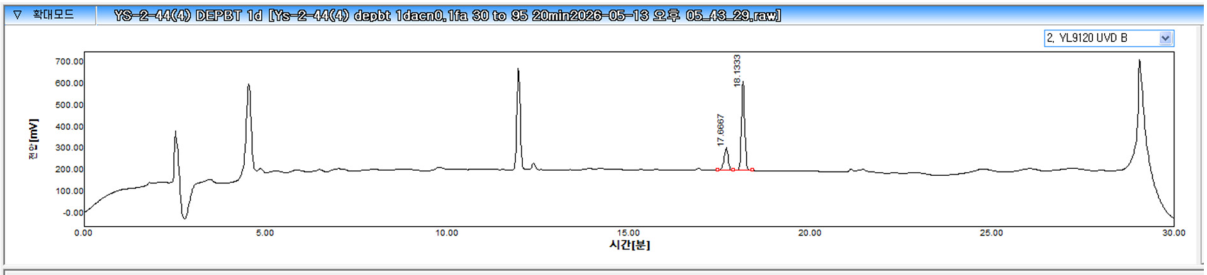

#	피크이름	RT[분]	면적[mV*sec]	면적비[%]	높이[mV]	높이비[%]	농도	농도비[%]	형태	폭[초]	반치폭[초]	이론단수	분리도	Tailing Factor	사용자 정의1	사용자 정의2
1		17,6667	809,3396	23,69	104,8018	20,25	0,000000	0,00	PV	9,23	6,82	210880,8	0,0000	0,9160	0,000000	0,000000
2		18,1333	2606,3623	76,31	412,8013	79,75	0,000000	0,00	VB	9,12	5,83	227719,5	3,0513	1,0519	0,000000	0,000000
합계			3415,7019		517,6031		0,000000									

Compound	Retention time (min)	Area (%)	Ratio
**S2**	18.13	76.31	1.00
**2a**	17.67	23.69	0.31
**2b**	-	-	-

Entry 9. PyBOP (5.0 eq.), DIPEA (5.0 eq.), DMF (1 mM), slow addition for 12 h

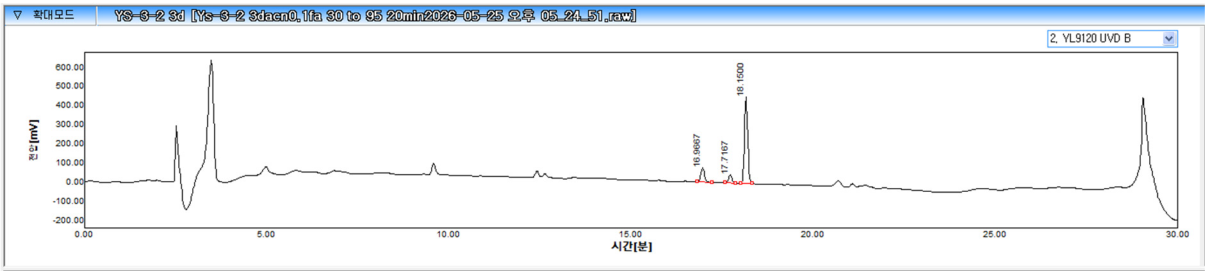

#	피크이름	RT[분]	면적[mV*sec]	면적비[%]	높이[mV]	높이비[%]	농도	농도비[%]	형태	폭[초]	반치폭[초]	이론단수	분리도	Tailing Factor	사용자 정의1	사용자 정의2
1		16,9667	488,0354	13,77	73,0317	12,84	0,000000	0,00	BB	8,53	6,13	227989,2	0,0000	1,0260	0,000000	0,000000
2		17,7167	278,5173	7,86	43,0623	7,57	0,000000	0,00	PB	8,78	6,13	234457,7	5,1995	1,0662	0,000000	0,000000
3		18,1500	2778,7471	78,38	452,7505	79,59	0,000000	0,00	BB	8,35	5,74	272342,0	3,0359	1,0719	0,000000	0,000000
합계			3545,2998		568,8444		0,000000									

Compound	Retention time (min)	Area (%)	Ratio
**S2**	18.15	78.38	1.00
**2a**	17.72	7.86	0.10
**2b**	16.97	13.77	0.18

Copies of NMR Spectra

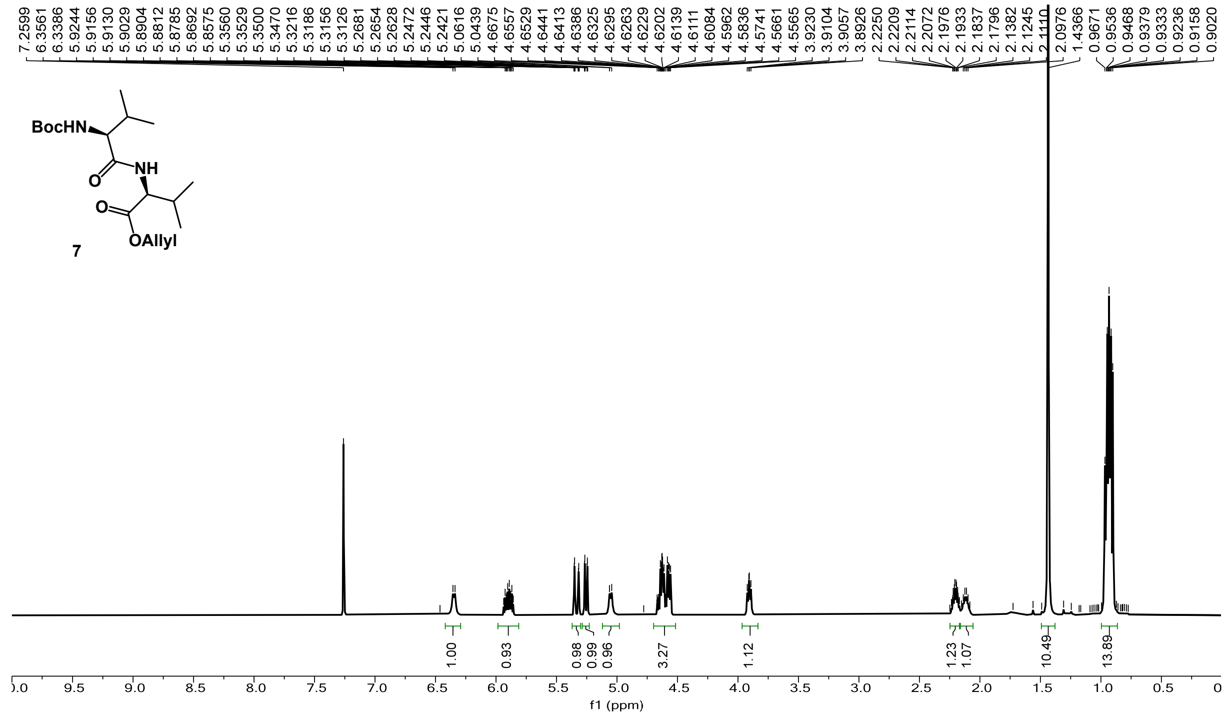

▲ ^{1}H NMR of **7** (500 MHz, $CDCl_3$)

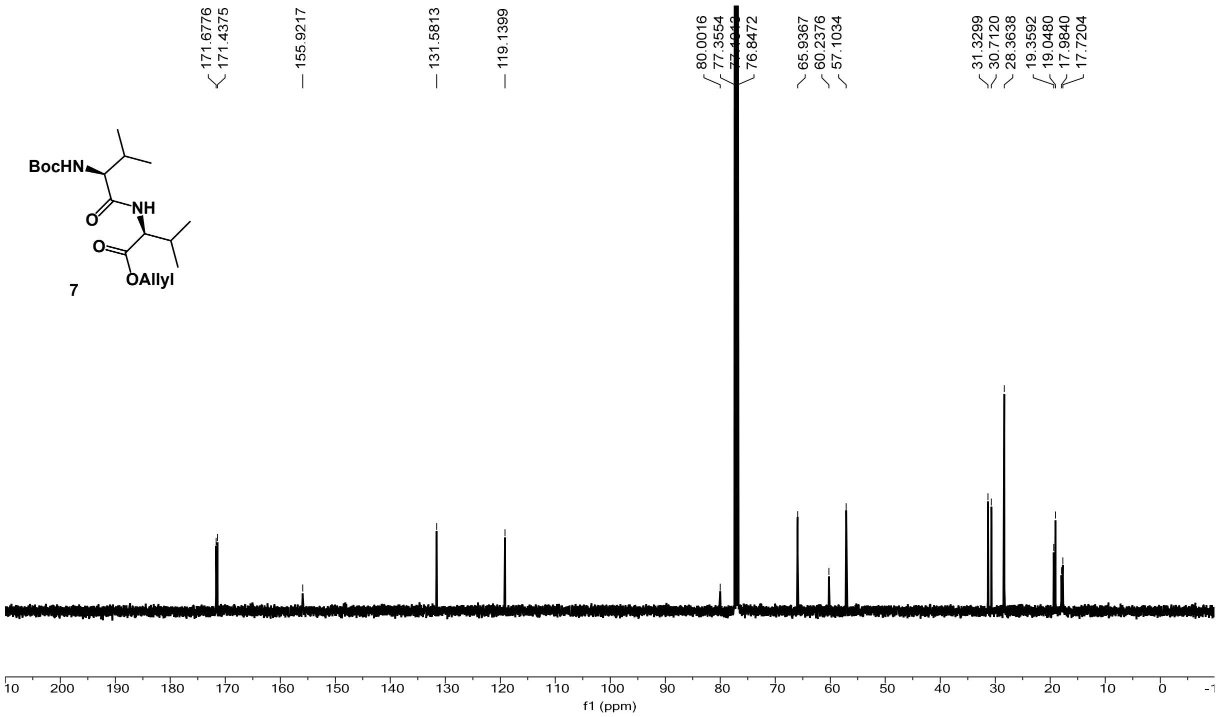

▲ ^{13}C NMR of **7** (126 MHz, $CDCl_3$)

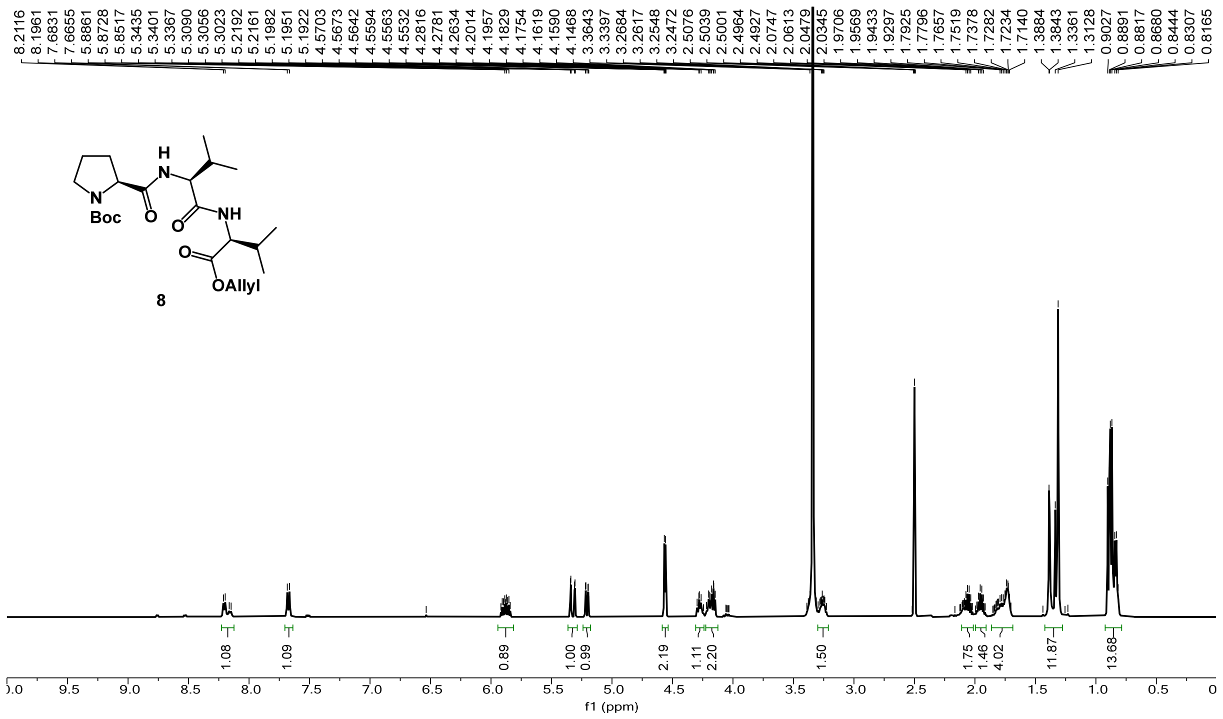

▲ ^{1}H NMR of **8** (500 MHz, DMSO-d_6)

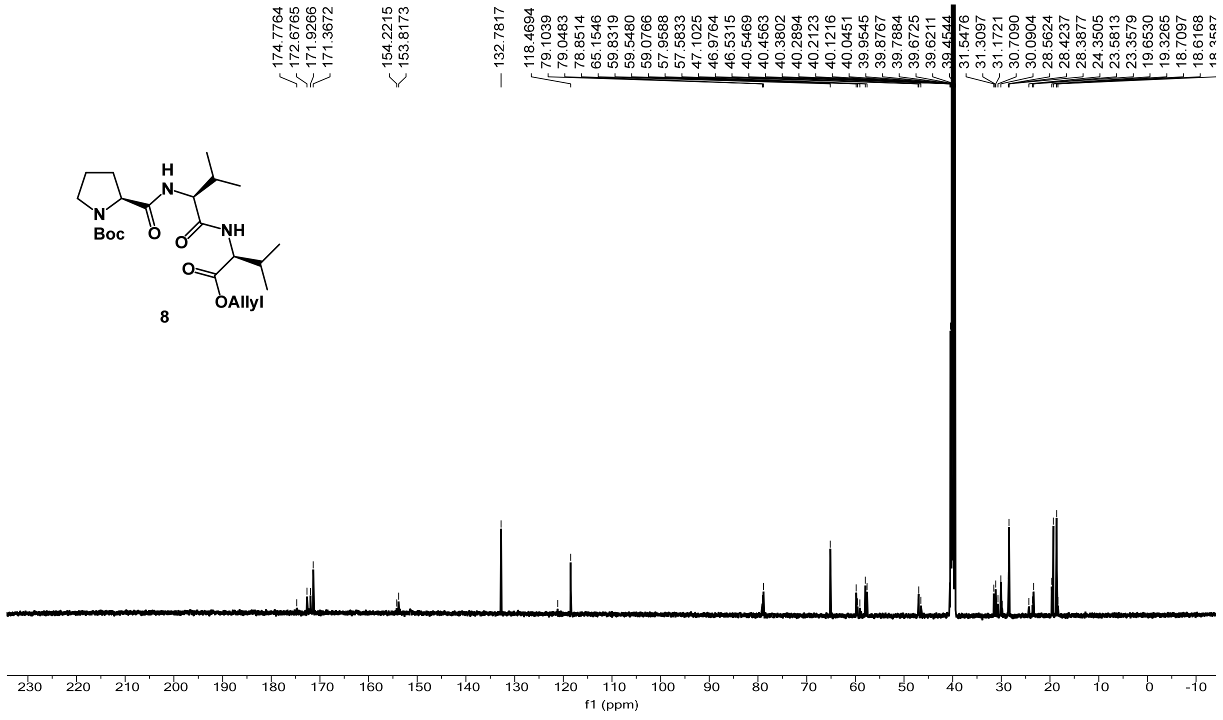

▲ ^{13}C NMR of **8** (126 MHz, DMSO-d_6)

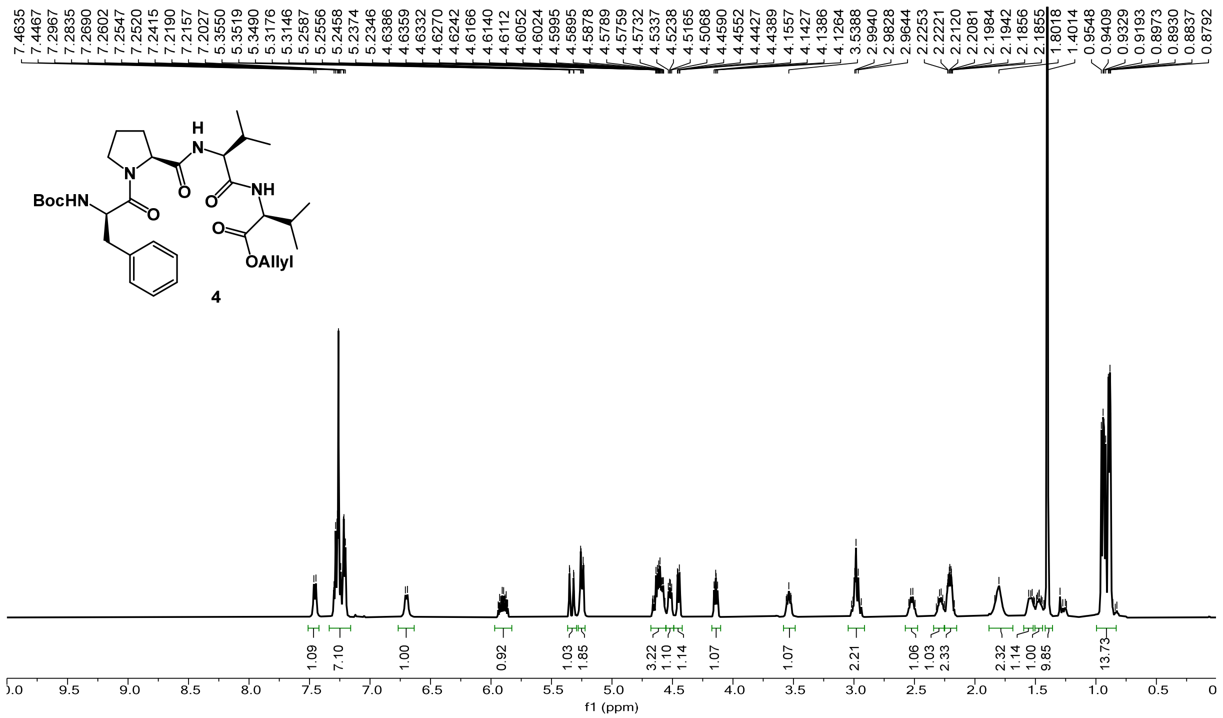

▲ ^{1}H NMR of **4** (500 MHz, $CDCl_3$)

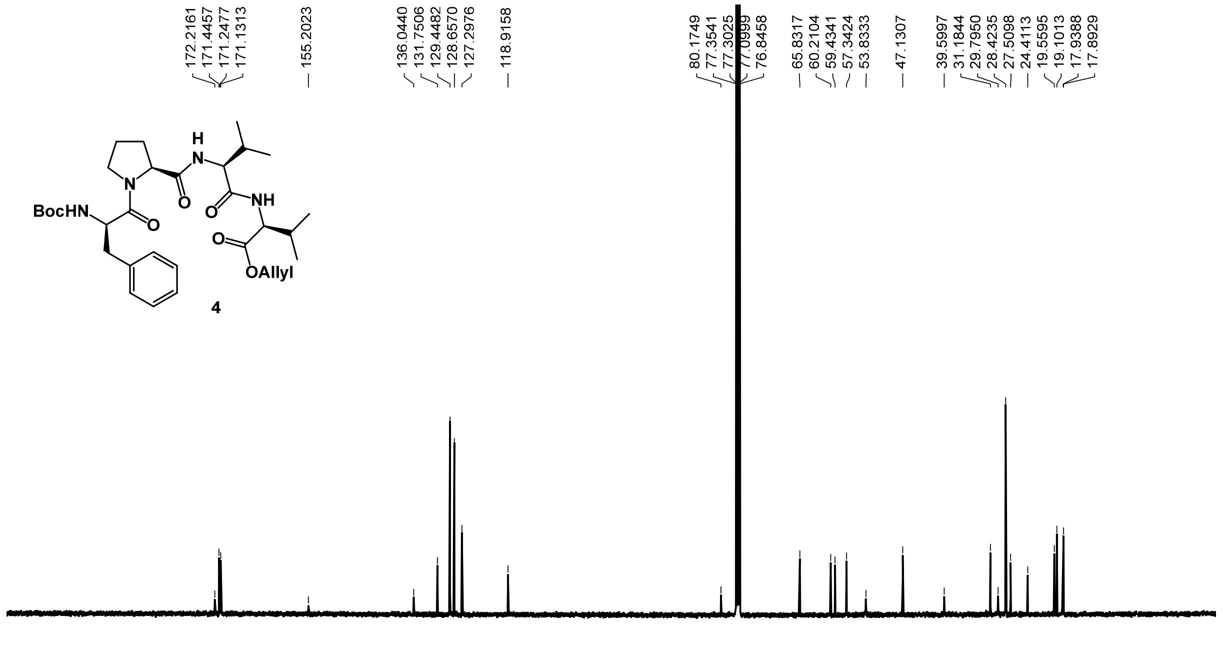

▲ ^{13}C NMR of **4** (126 MHz, $CDCl_3$)

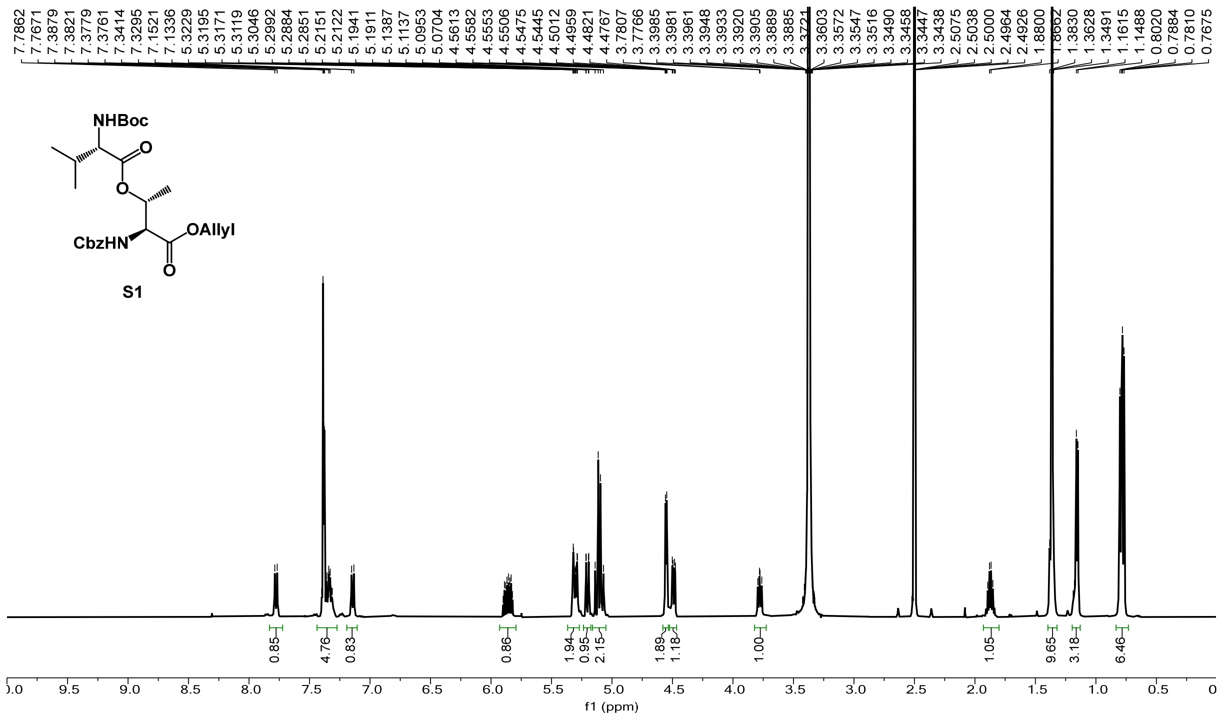

▲ ^{1}H NMR of **5** (500 MHz, DMSO-d_6)

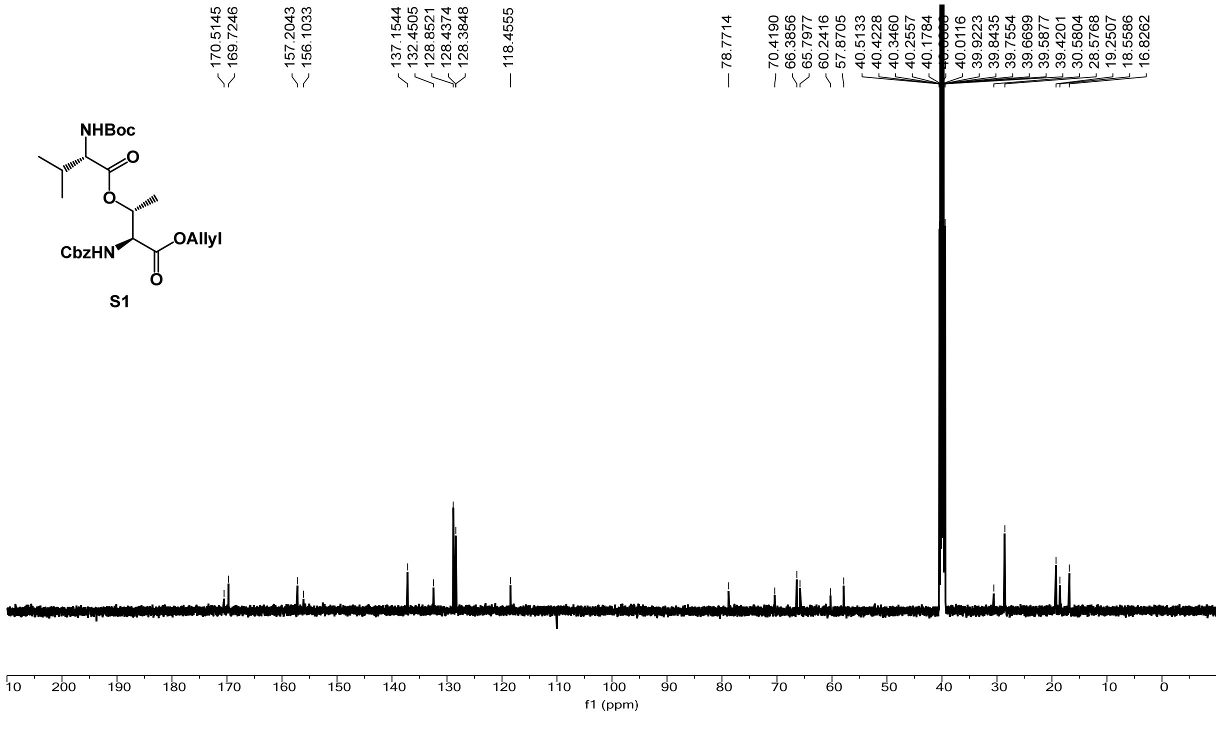

▲ ^{13}C NMR of **5**(126 MHz, DMSO-d_6)

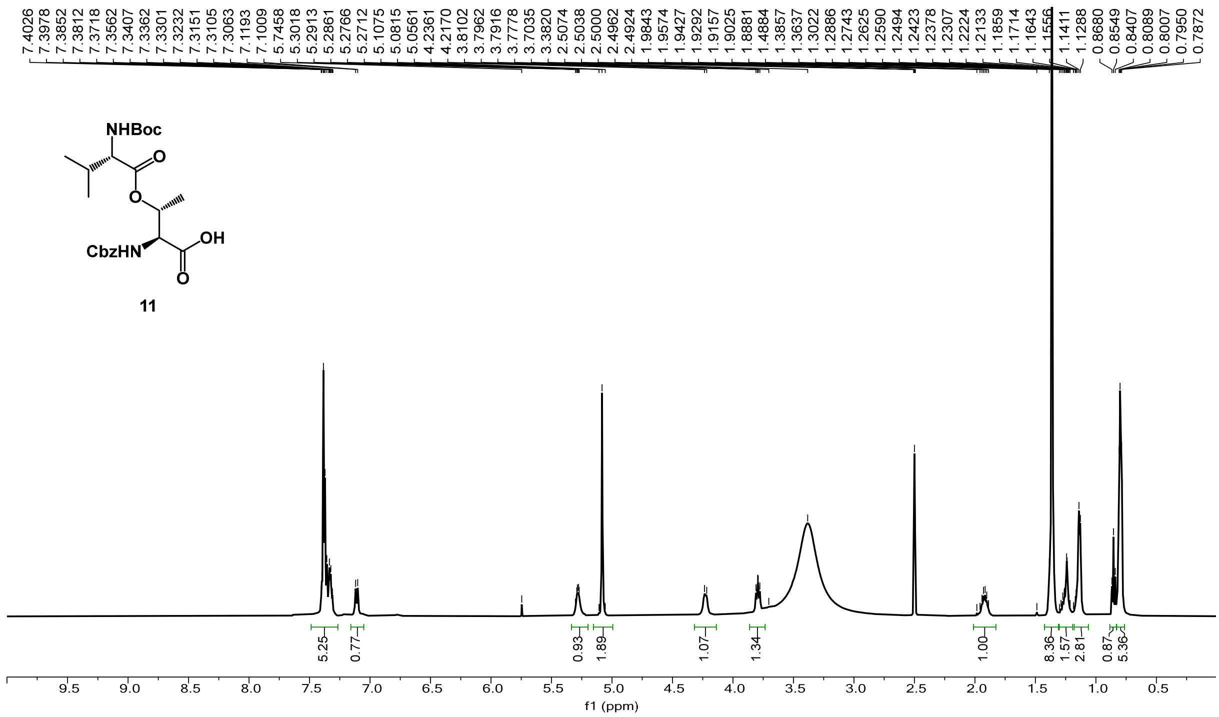

▲ ^{1}H NMR of **11** (500 MHz, DMSO-d_6)

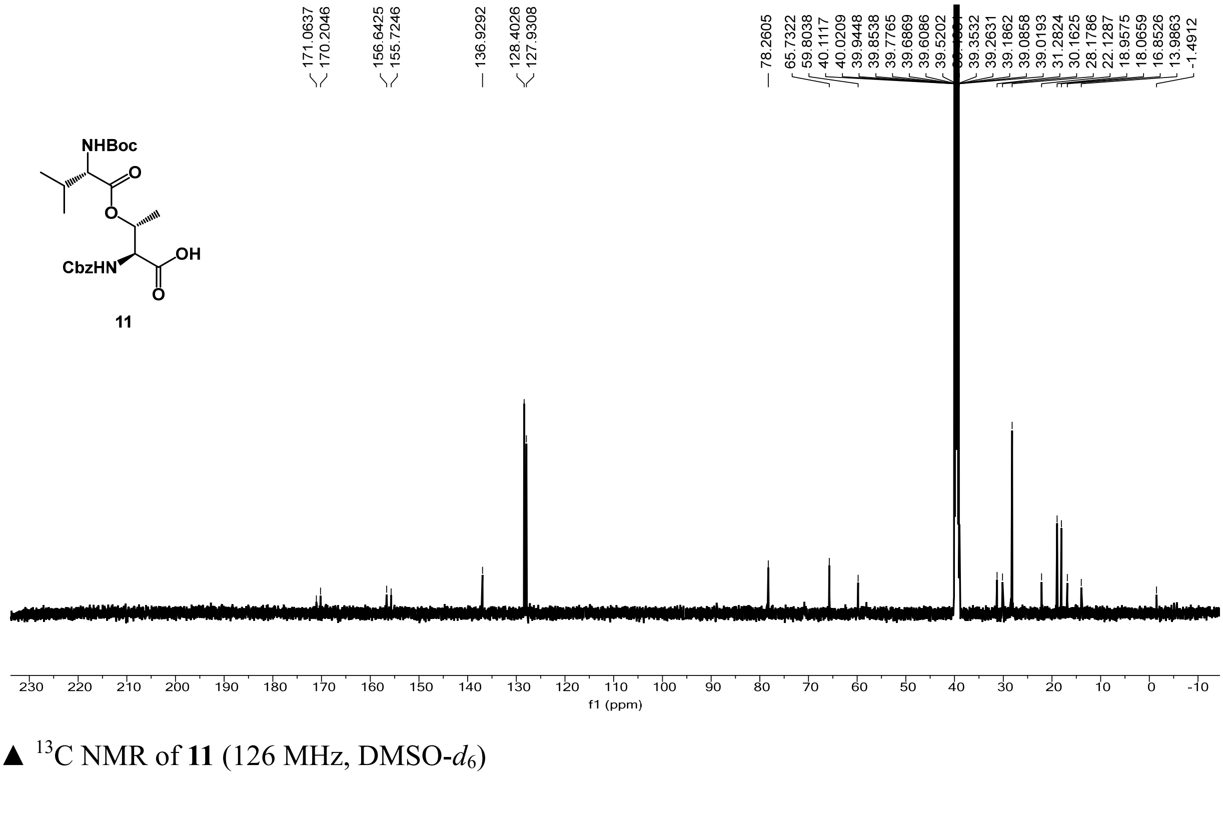

▲ ^{13}C NMR of **11** (126 MHz, DMSO-d_6)

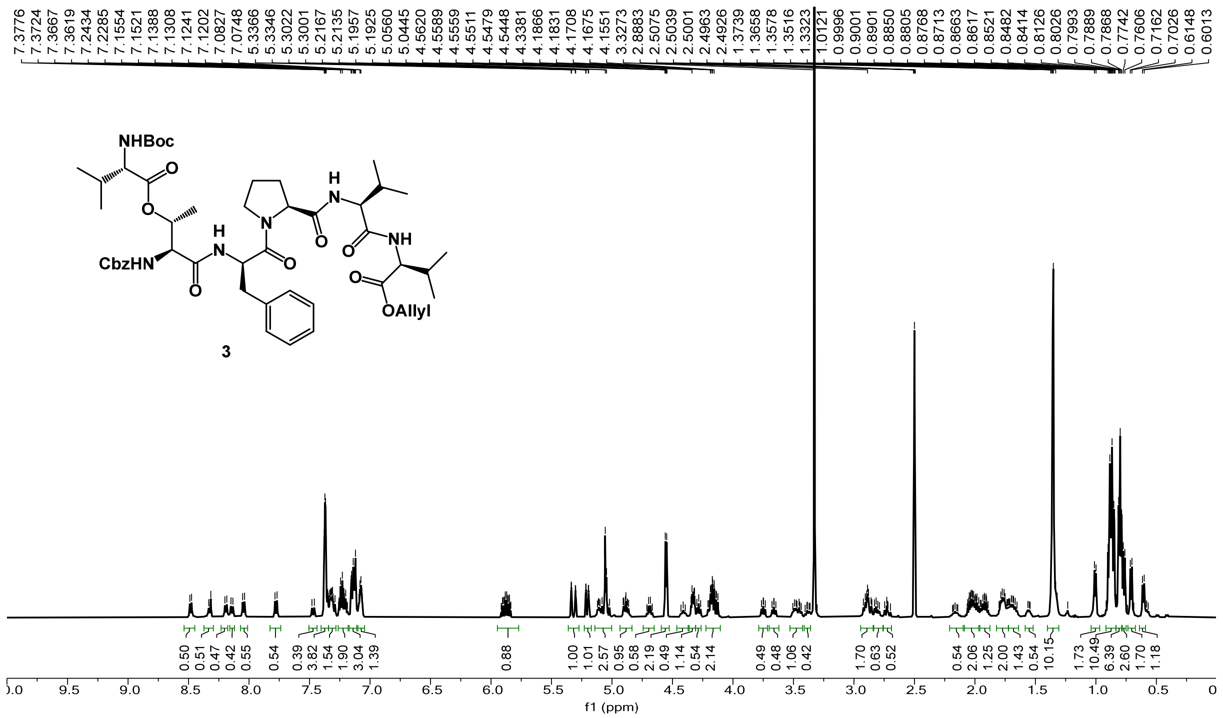

▲ ^{1}H NMR of **3** (500 MHz, DMSO-d_6)

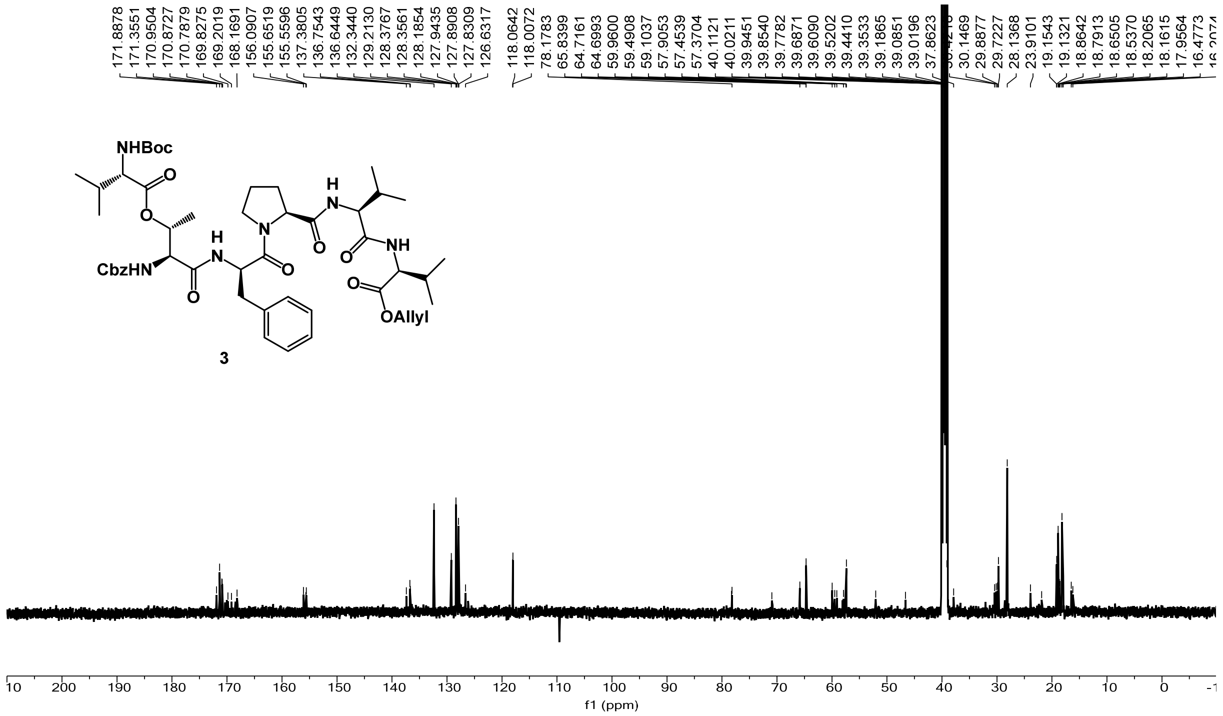

▲ ^{13}C NMR of **3** (126 MHz, DMSO-d_6)

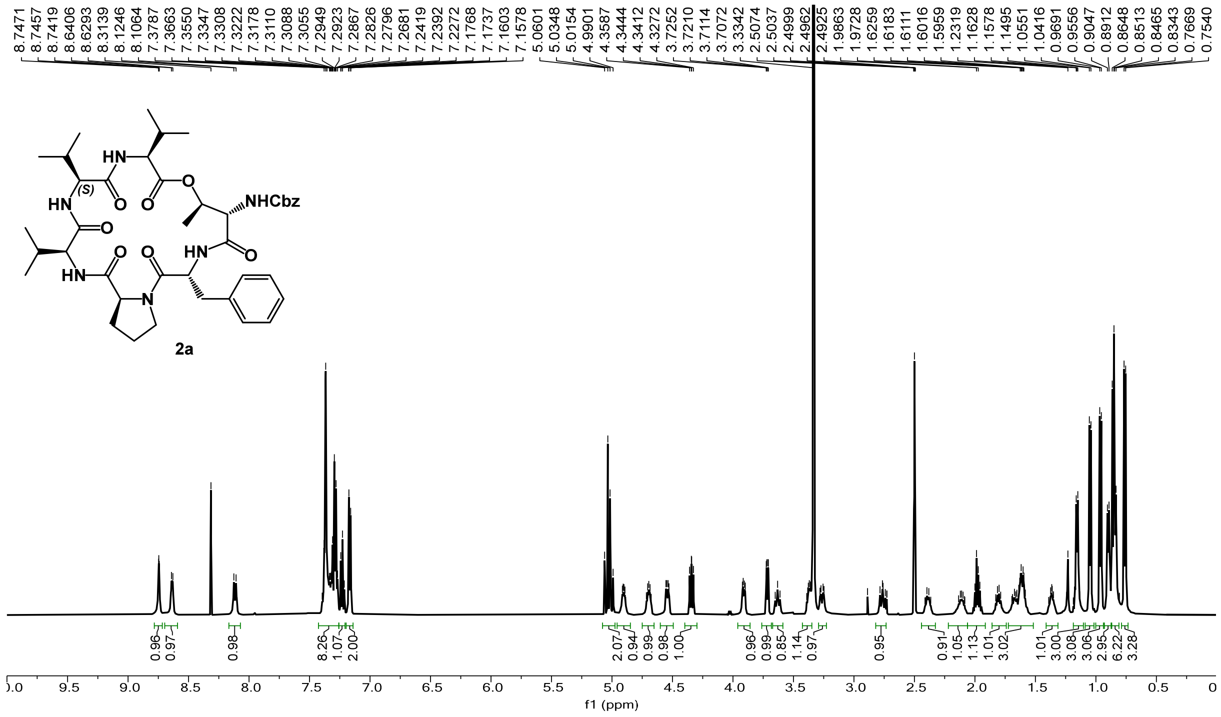

▲ ^{1}H NMR of **2a** (500 MHz, DMSO-*d*6)

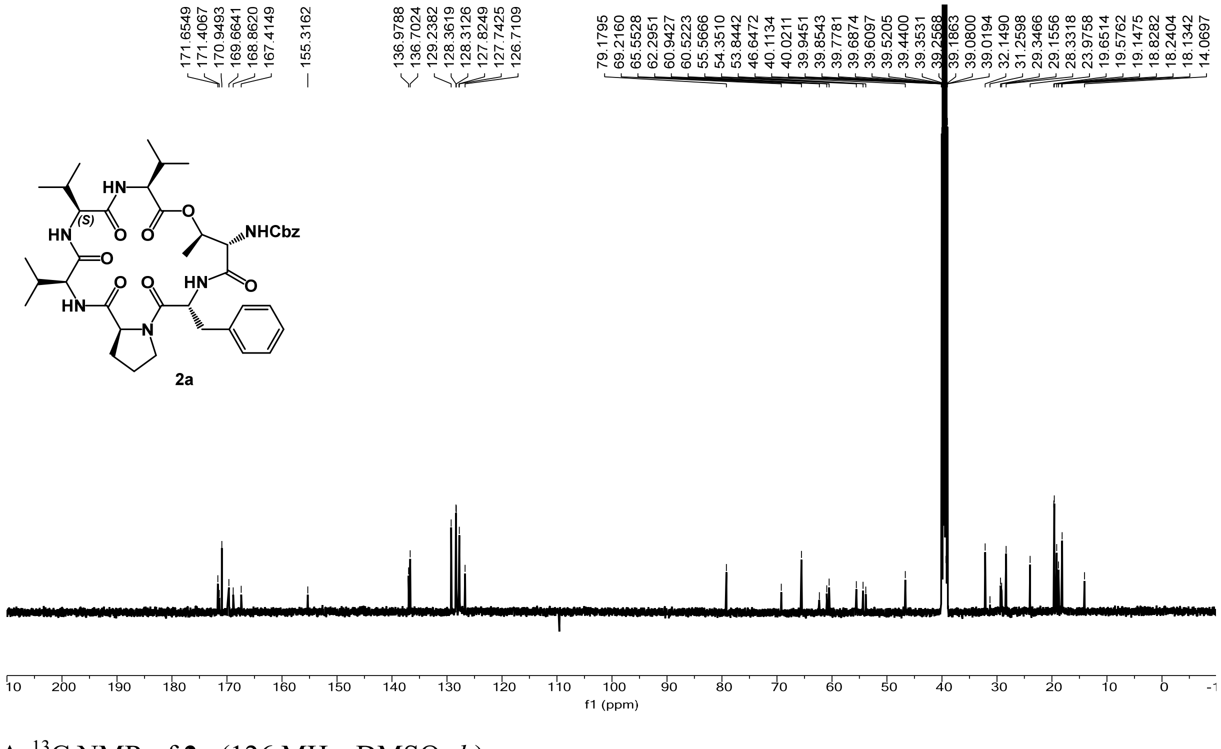

▲ ^{13}C NMR of **2a** (126 MHz, DMSO-*d*6)

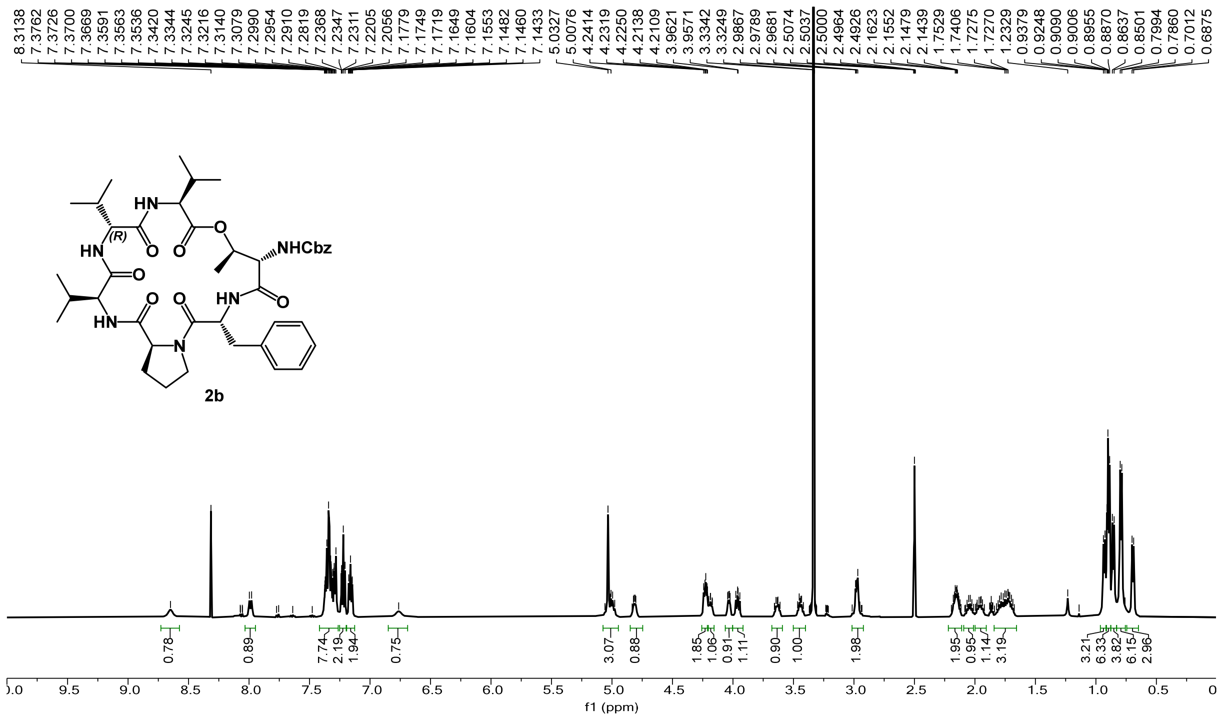

▲ ^{1}H NMR of **2b** (500 MHz, DMSO-d_6)

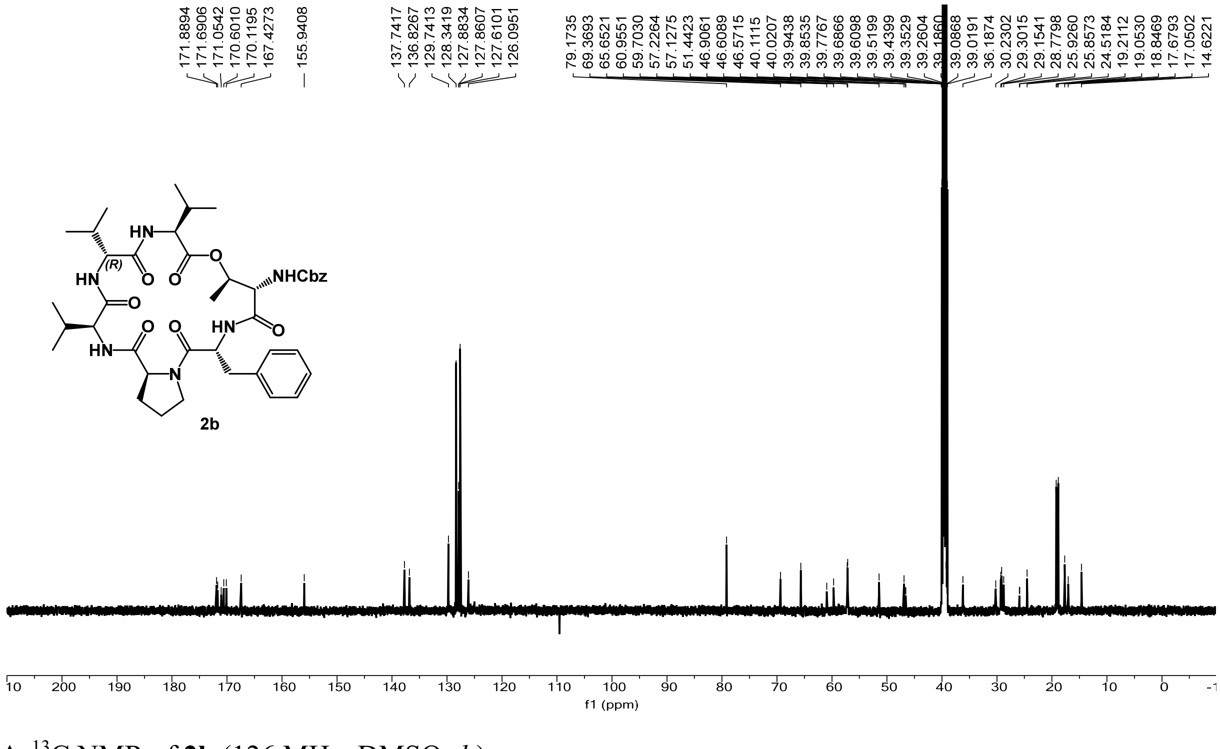

▲ ^{13}C NMR of **2b** (126 MHz, DMSO-d_6)

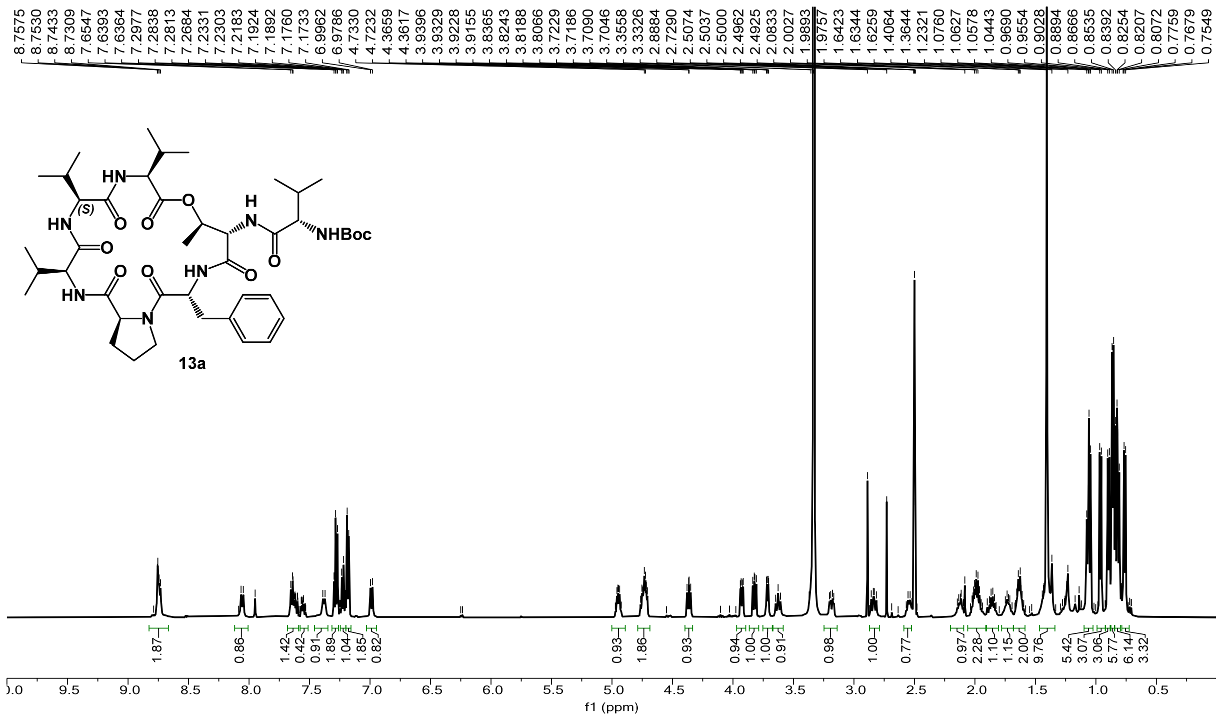

▲ ^{1}H NMR of **13a** (500 MHz, $CDCl_3$)

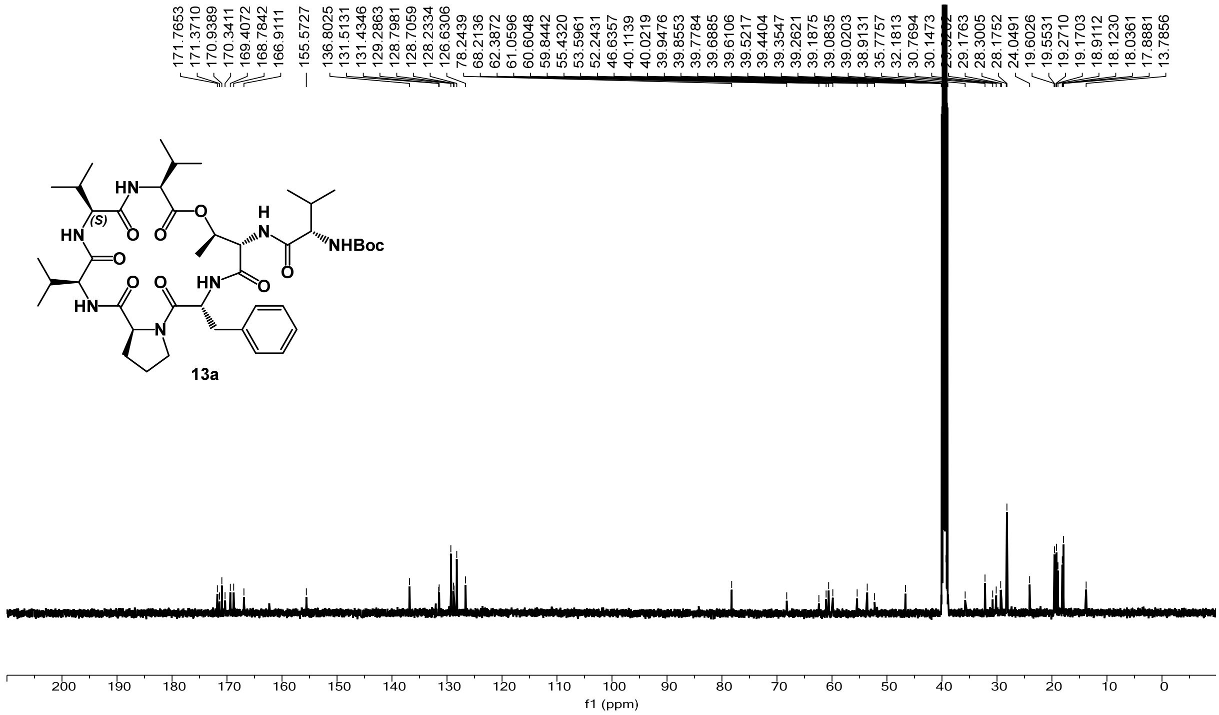

▲ ^{13}C NMR of **13a** (126 MHz, $CDCl_3$)

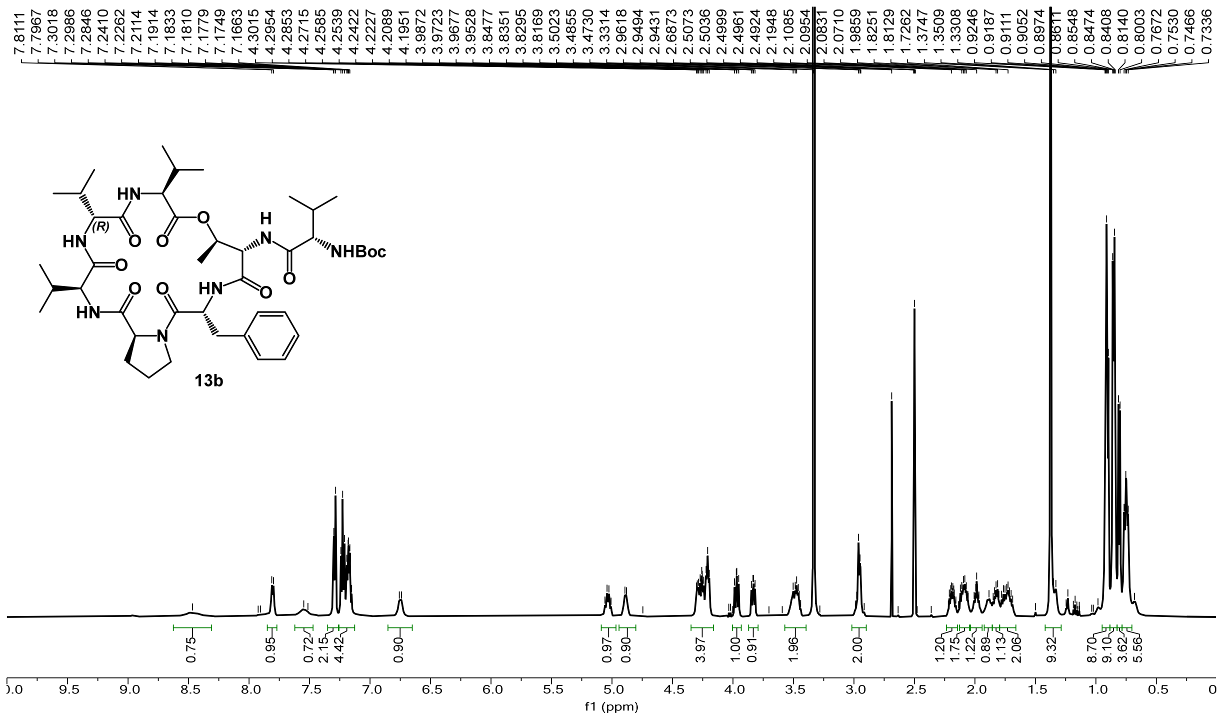

▲ ^{1}H NMR of **13b** (500 MHz, $CDCl_3$)

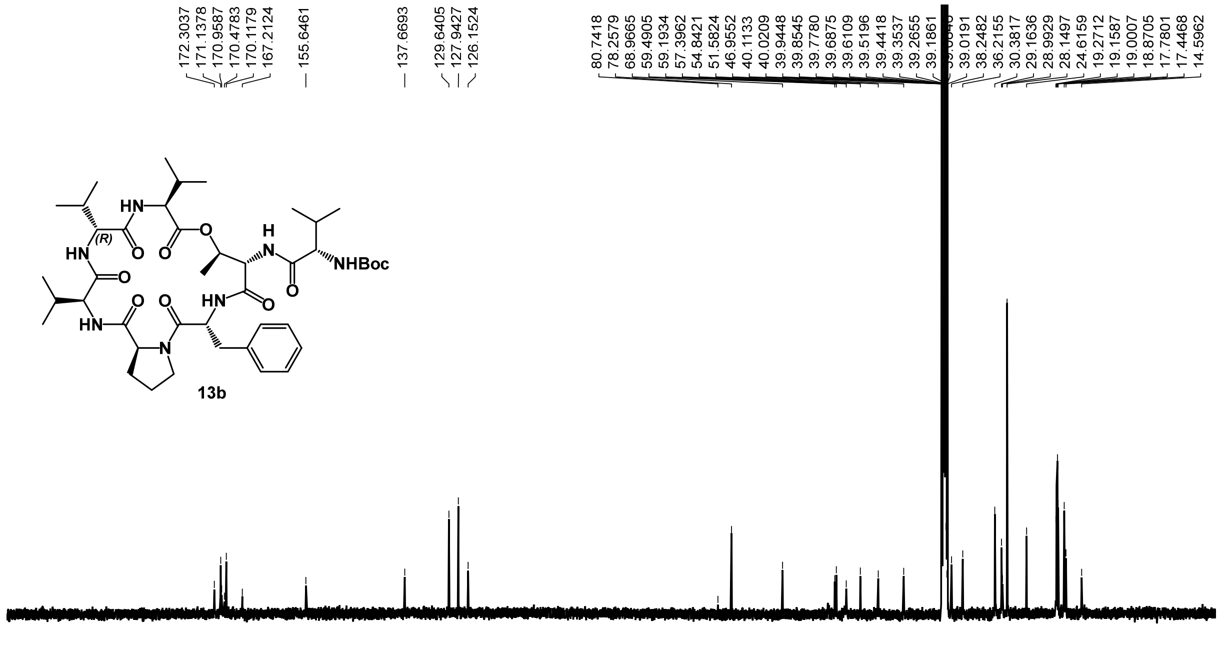

▲ ^{13}C NMR of **13b** (126 MHz, $CDCl_3$)

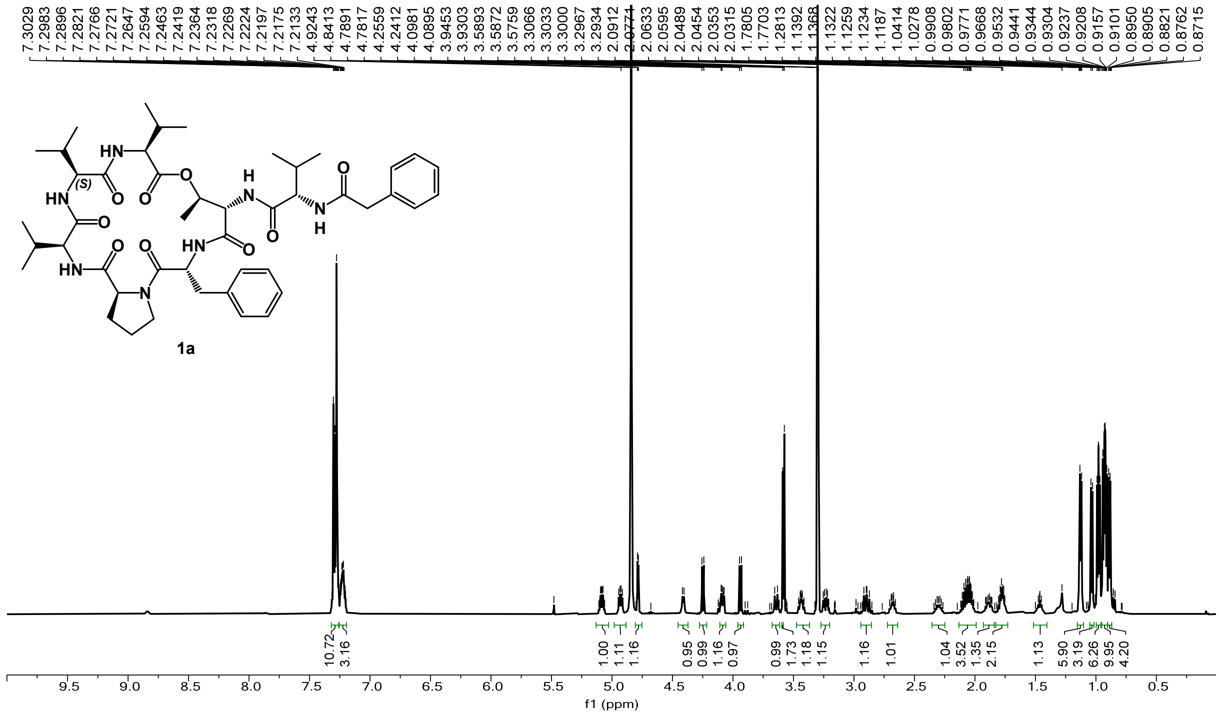

▲ ^{1}H NMR of Xentrivalpeptide A (**1a**, 500 MHz, CD_3OD)

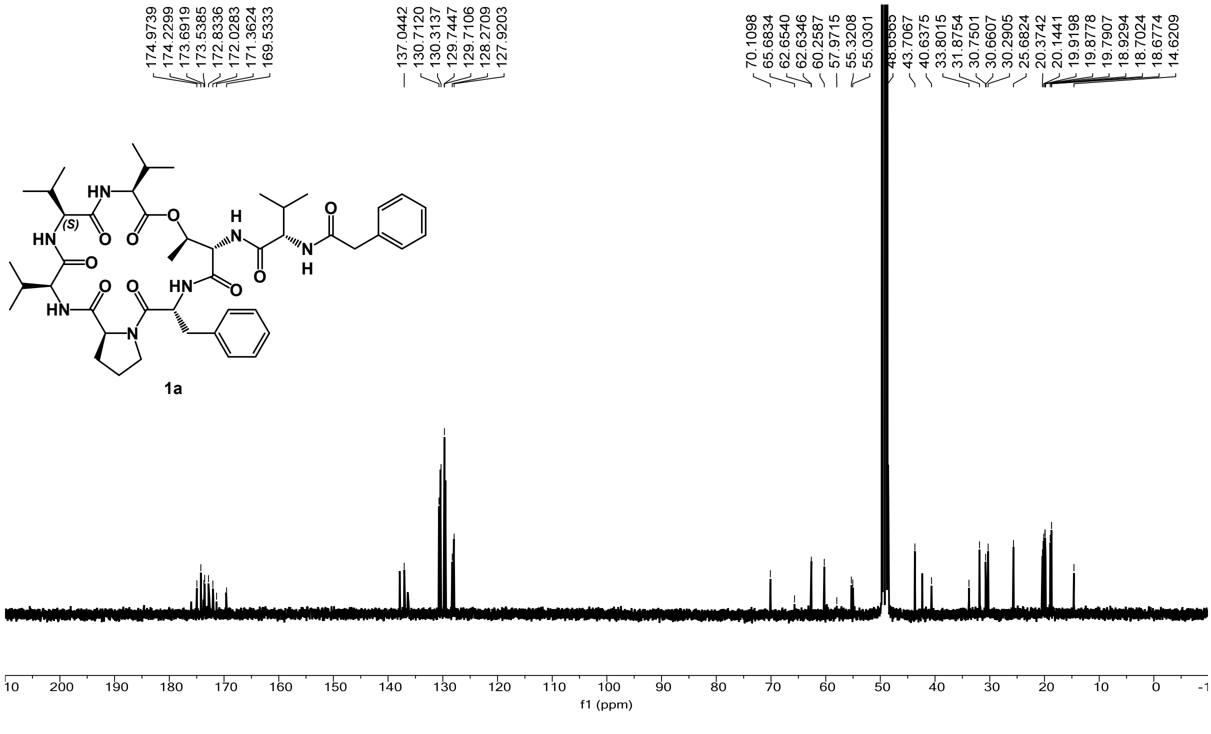

▲ ^{13}C NMR of Xentrivalpeptide A (**1a**, 126 MHz, CD_3OD)

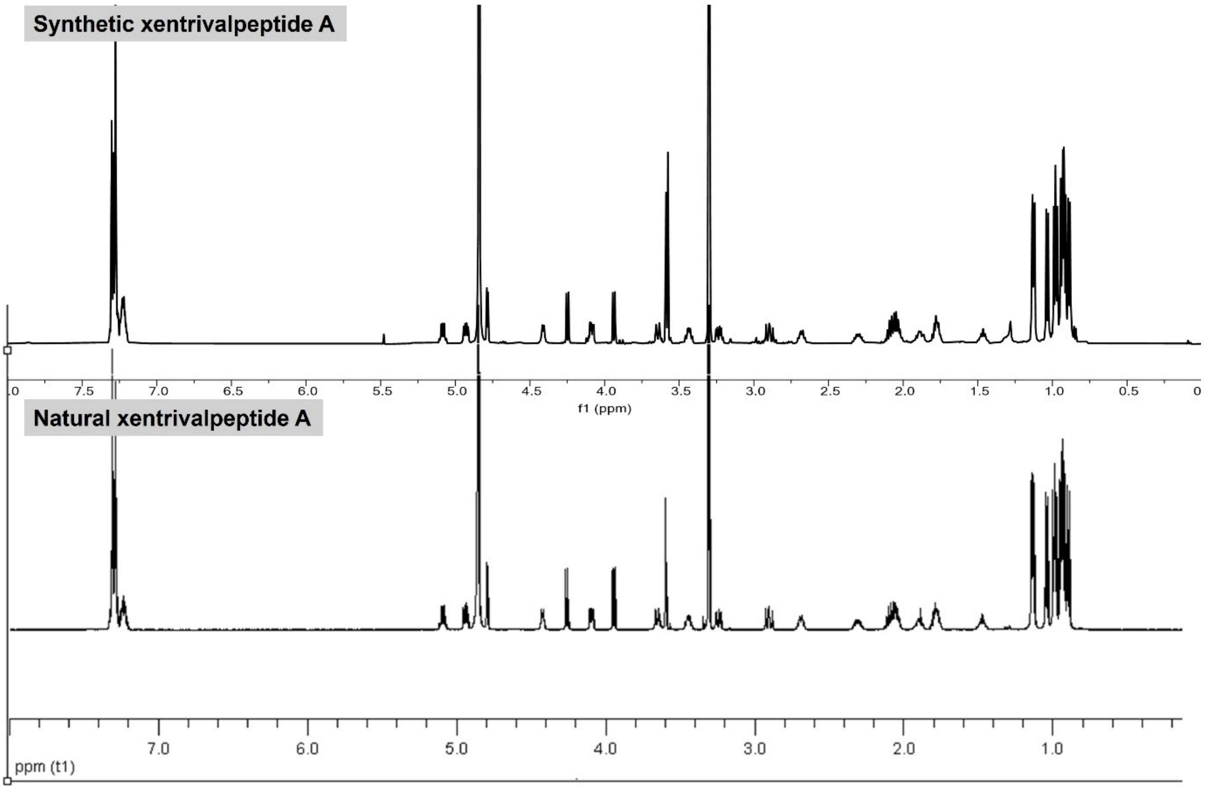

▲ ^{1}H NMR comparison of natural and synthetic xentrivalpeptide A (**1a**, CD_3OD)

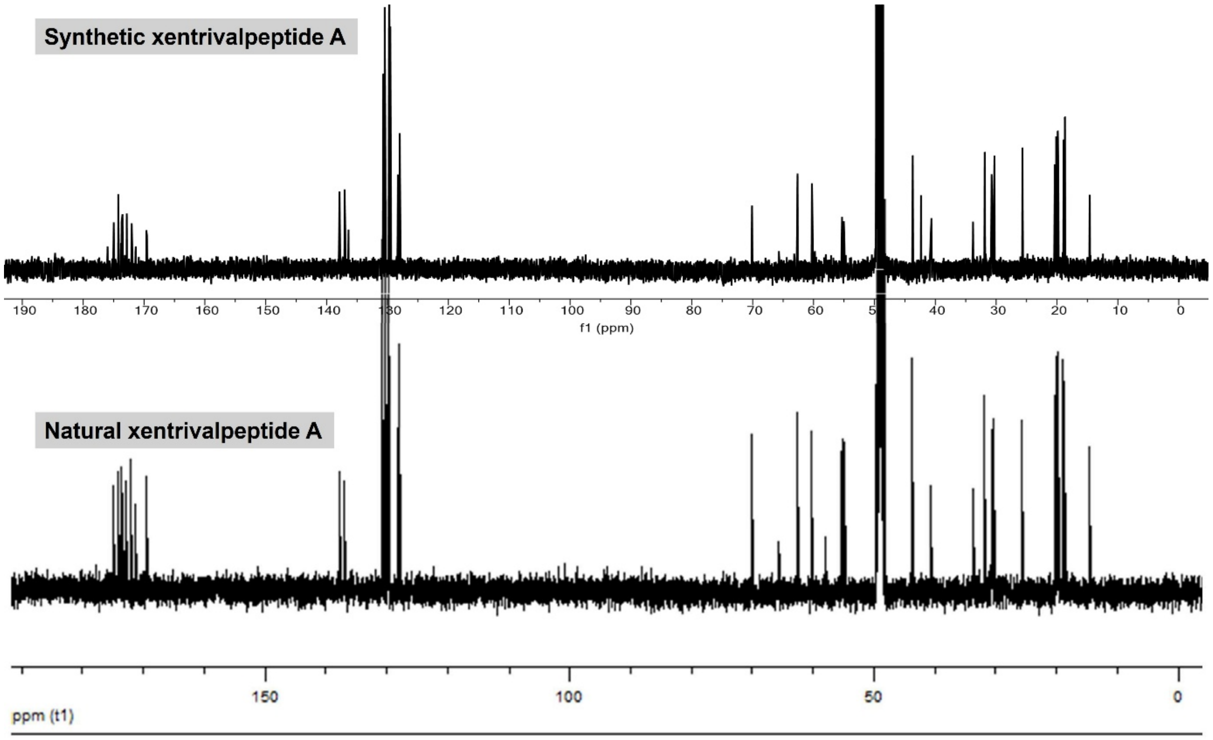

▲ ^{13}C NMR comparison of natural and synthetic xentrivalpeptide A (**1a**, CD_3OD)

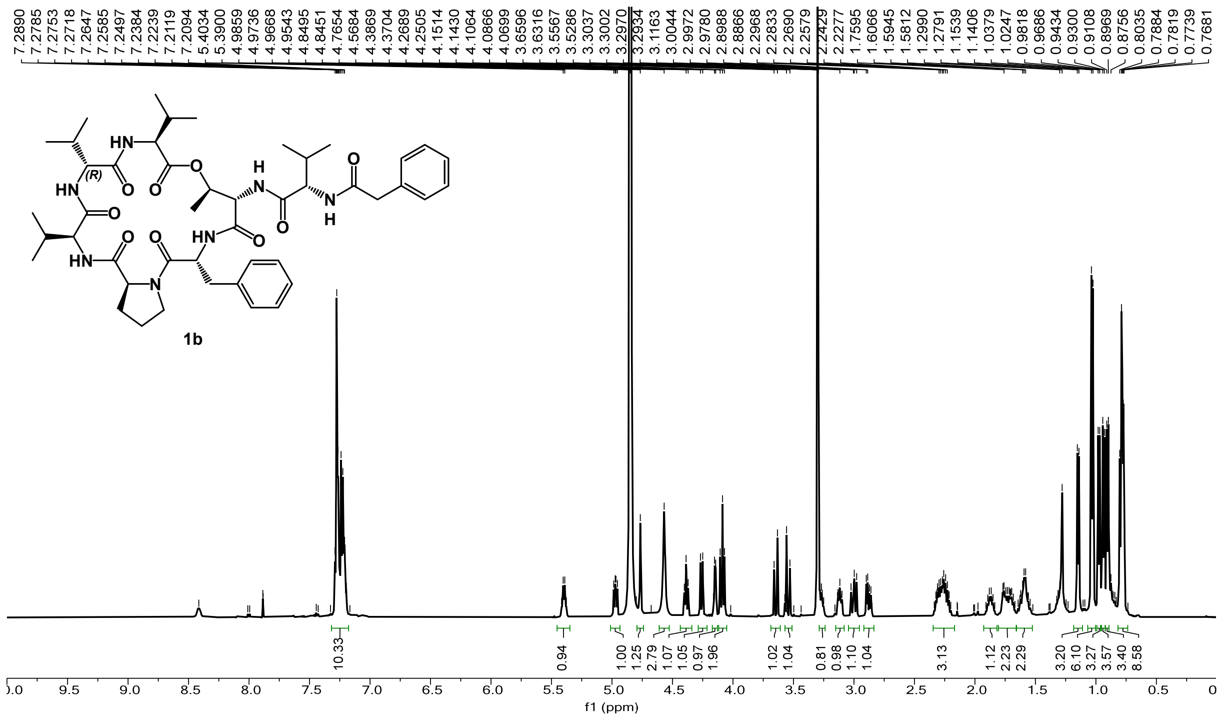

▲ ^{1}H NMR of 6α-*epi*-Xentrivalpeptide A (**1b**, 500 MHz, CD_3OD)

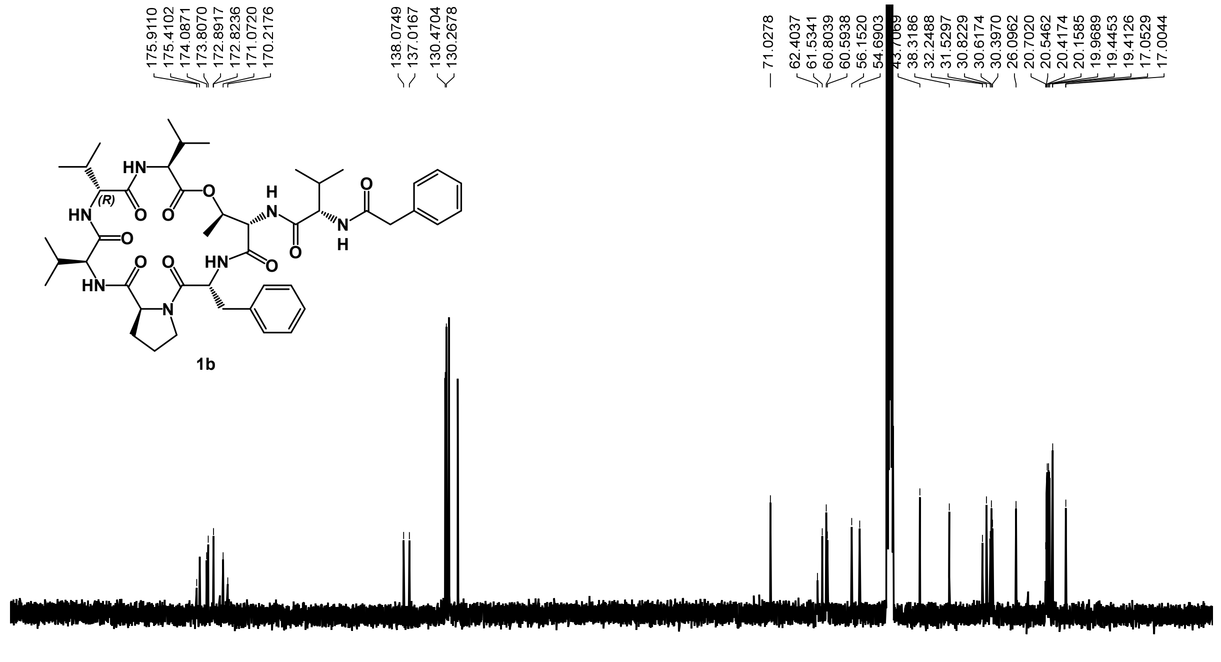

▲ ^{13}C NMR of 6α-*epi*-Xentrivalpeptide A (**1b**, 126 MHz, CD_3OD)

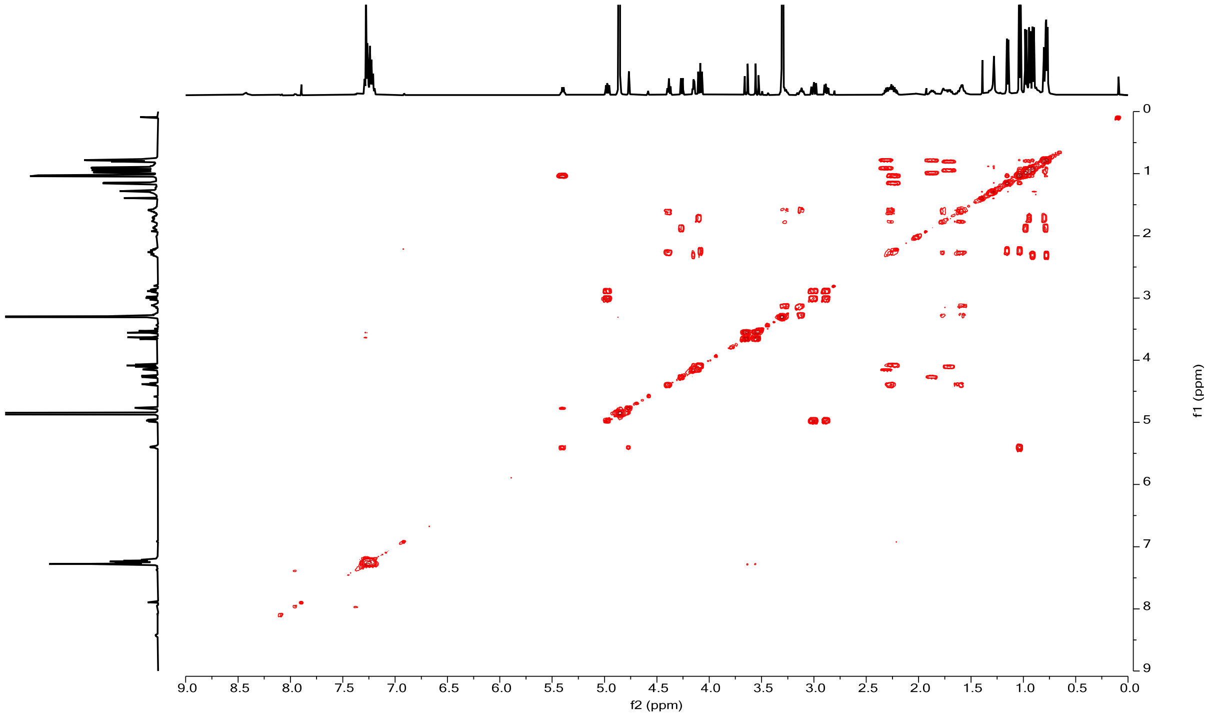

▲ COSY of 6α-*epi*-Xentrivalpeptide A (**1b**, 500 MHz, CD_3OD)

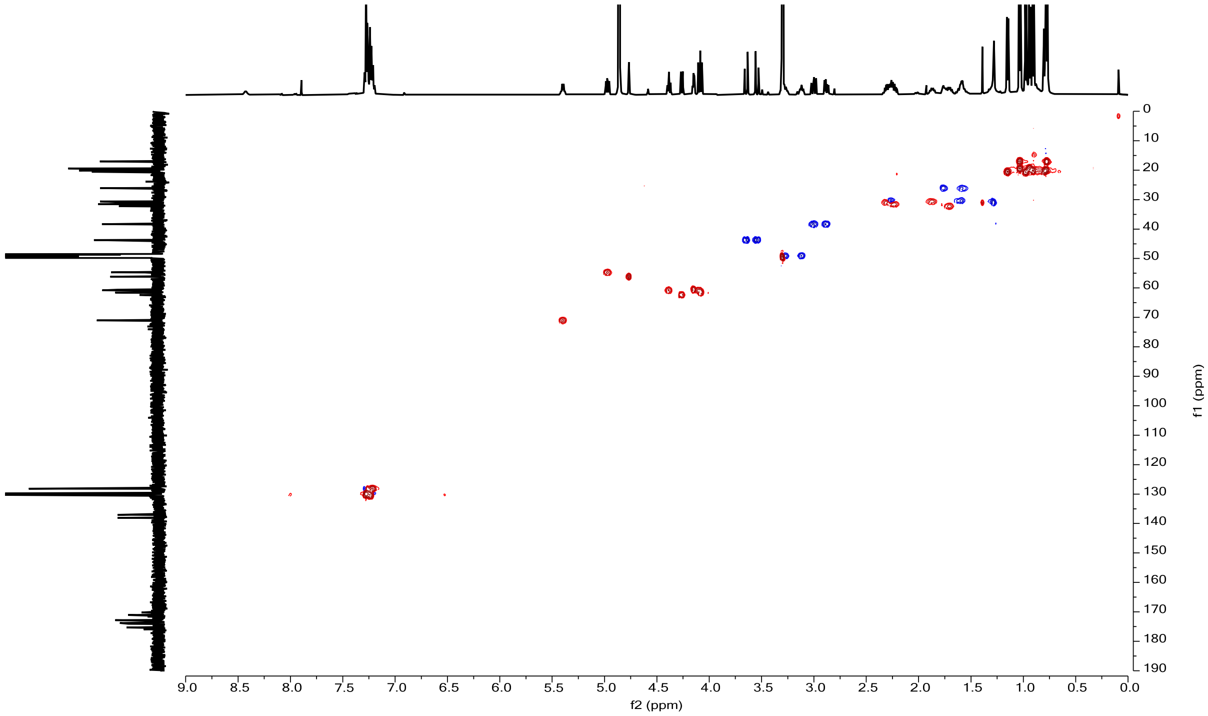

▲ HSQC of 6α-*epi*-Xentrivalpeptide A (**1b**, 126 MHz, CD_3OD)

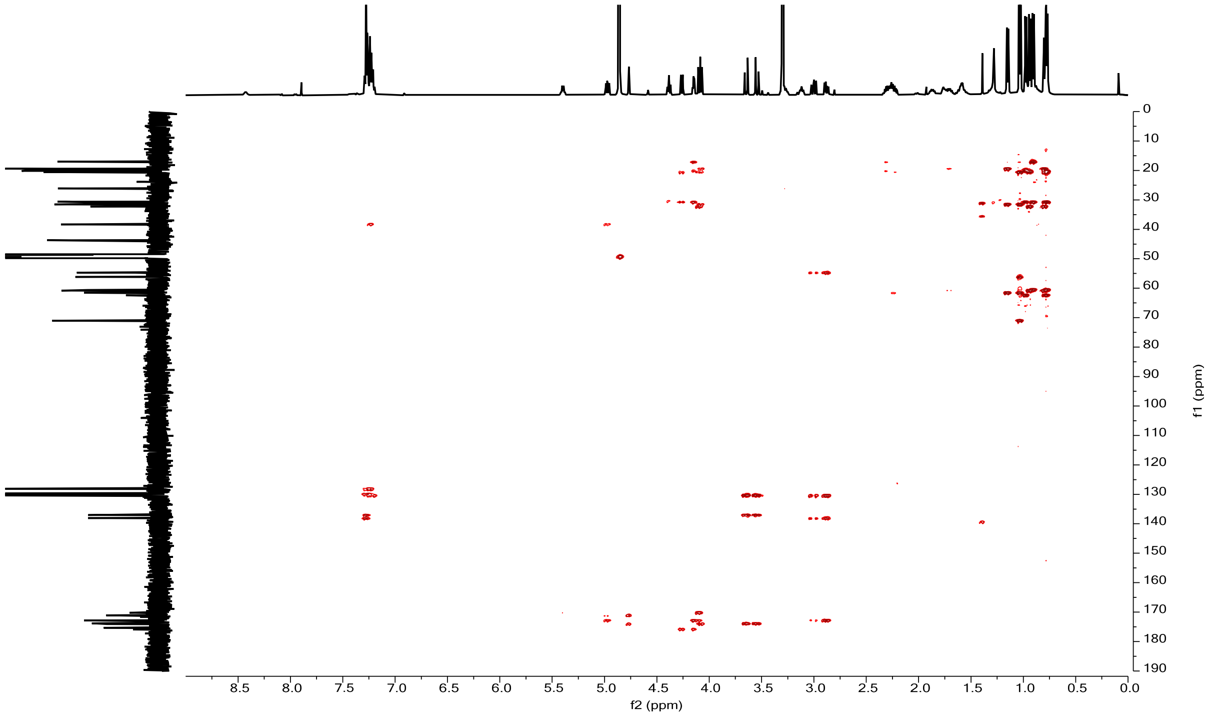

▲ HMBC of 6α-*epi*-Xentrivalpeptide A (**1b**, 500 MHz, CD_3OD)

References

1. Zhou, Q.; Dowling, A.; Heide, H.; Wöhnert, J.; Brandt, U.; Baum, J.; Ffrench-Constant, R.; Bode, H.B. Xentrivalpeptides A-Q: Depsipeptide Diversification in Xenorhabdus. *J. Nat. Prod.* **2012**, *75*, 1717–1722, doi:10.1021/np300279g.
